# Top-Down Feedback in an HMAX-Like Cortical Model of Object Perception Based on Hierarchical Bayesian Networks and Belief Propagation

**DOI:** 10.1371/journal.pone.0048216

**Published:** 2012-11-05

**Authors:** Salvador Dura-Bernal, Thomas Wennekers, Susan L. Denham

**Affiliations:** 1 Department of Physiology and Pharmacology, State University of New York Downstate Medical Center, Brooklyn, New York, United States of America; 2 Cognition Institute, University of Plymouth, Plymouth, Devon, United Kingdom; Max Planck Institute for Human Cognitive and Brain Sciences, Germany

## Abstract

Hierarchical generative models, such as Bayesian networks, and belief propagation have been shown to provide a theoretical framework that can account for perceptual processes, including feedforward recognition and feedback modulation. The framework explains both psychophysical and physiological experimental data and maps well onto the hierarchical distributed cortical anatomy. However, the complexity required to model cortical processes makes inference, even using approximate methods, very computationally expensive. Thus, existing object perception models based on this approach are typically limited to tree-structured networks with no loops, use small toy examples or fail to account for certain perceptual aspects such as invariance to transformations or feedback reconstruction. In this study we develop a Bayesian network with an architecture similar to that of HMAX, a biologically-inspired hierarchical model of object recognition, and use loopy belief propagation to approximate the model operations (selectivity and invariance). Crucially, the resulting Bayesian network extends the functionality of HMAX by including top-down recursive feedback. Thus, the proposed model not only achieves successful feedforward recognition invariant to noise, occlusions, and changes in position and size, but is also able to reproduce modulatory effects such as illusory contour completion and attention. Our novel and rigorous methodology covers key aspects such as learning using a layerwise greedy algorithm, combining feedback information from multiple parents and reducing the number of operations required. Overall, this work extends an established model of object recognition to include high-level feedback modulation, based on state-of-the-art probabilistic approaches. The methodology employed, consistent with evidence from the visual cortex, can be potentially generalized to build models of hierarchical perceptual organization that include top-down and bottom-up interactions, for example, in other sensory modalities.

## Introduction

### The Bayesian Brain Hypothesis

Experimental evidence shows that feedback originating in higher-level areas, such as V4, inferotemporal (IT) cortex, lateral occipital complex (LOC) or middle temporal (MT) cortex with bigger and more complex receptive fields, can modify and shape V1 responses, accounting for contextual or extra-classical receptive field effects [1]–[3].

While there is relative agreement that feedback connections play a role in integrating global and local information from different cortical regions to generate an integrated percept [4], [5], several differing approaches have attempted to explain the underlying mechanisms. Generative models and the Bayesian brain hypothesis [6] provide a framework that can quantitatively model the interaction between prior knowledge and sensory evidence, in order to represent the physical and statistical properties of the environment. The Bayesian brain concept is not just limited to the sensory cortex, but has also been applied to motor cortex [7] and other brain regions such as the hippocampus [8], [9].

Overall, increasing evidence supports the proposal that Bayesian inference provides a theoretical framework that maps well onto cortical connectivity, explains both psychophysical and neurophysiological results, and can be used to build biologically plausible models of brain function [6], [10]–[12]. Within this framework, Bayesian networks and belief propagation provide a rigorous mathematical foundation for these principles. Belief propagation has been found to be particularly well-suited to neural implementation, due to its hierarchical distributed organization and homogeneous internal structure and operations [5], [13]–[16].

### Current Limitations

However, modelling cortical perceptual processes using this framework poses a number of problems. First of all, the main drawback inherent in belief propagation is its great computational cost in terms of speed and memory. The number of operations and the required memory grows exponentially with the number of parents of each node. Additionally, for networks with loops, such as those that arise when modelling the large-scale cortical connectivity, exact inference methods are intractable. Approximate solutions can be found using sampling methods [5], [17], [18], variational methods [6], [19], [20] or loopy belief propagation [13], [21], although these can also be very demanding as they usually require several iterations to converge.

For this reason, much of the research effort in the field focuses on strategies to optimize loopy belief propagation: reducing the complexity to generate messages [22]–[25]; grouping nodes with similar properties, such as in lifted networks [26] or tilebased propagation [24]; or message-passing schedules for faster convergence, for example using bipartite graphs [23], [25] or residual belief propagation [27], [28]. However, many of these solutions require specific types of variables, for example having a Gaussian distribution, or specific graph topologies, such as single-layer Markov random fields, which are not compatible with the hierarchical architecture required for object perception.

The second problem arises when modelling invariance to object transformations using probabilistic inference in graphical models. A classic non-probablistic approach is to extend the classic work by Hubel and Wiesel on simple and complex cells to generate multi-stage Hubel-Wiesel architectures, such as the multilayer perceptron [29], convolutional neural networks (ConvNets) [30] or the HMAX model [31]. These architectures alternate feature selectivity layers with pooling or subsampling layers, where the pooling operation is typically the max or average function.

The Hierarchical Temporal Memory (HTM) [13] attempts to replicate this structure in probabilistic terms by defining nodes that contain features and sequences of features. Thus, simple and complex nodes are merged into a single node, which differs from the conventional definition of Bayesian node and so requires an adapted version of the belief propagation algorithm.

Recently, Convolutional Deep Belief Networks (CDBNs) have been proposed [32] to bridge the gap between Deep Belief Networks [17] and the multi-stage Hubel-Wiesel architecture. Deep Belief Networks consist of multiple layers of Restricted Boltzmann Machines, an undirected graphical model of binary variables, that can perform probabilistic inference using Gibbs sampling. CDBNs extend this model by incorporating a probabilistic *max-pooling* operation and weight sharing, enabling it to implement alternating selectivity and invariance layers and yielding state-of-the-art results in object recognition.

### Proposal

In this paper we first provide a description of HMAX, a biologically-inspired hierarchical model of object recognition, which our proposed model aims to reformulate in probabilistic terms and extend with feedback. We then provide an introduction to Bayesian networks and belief propagation, the modelling tools used in our approach. Afterwards, we describe the proposed model: a Bayesian network with discrete-valued variables that reproduces an HMAX-like architecture and employs loopy belief propagation to approximate the functionality of HMAX, i.e. selectivity and invariance in alternating layers. A detailed toy-example is then employed to explain how the model works and how the HMAX operations are approximated. The layerwise greedy learning algorithm, vital to achieve the desired functionality, are subsequently detailed.

The Methods section also includes a description of several approximations that simplify the belief propagation algorithm and allow it to run on large-scale Bayesian networks, such as the one proposed. The most important approximation consists of sampling the incoming messages to keep only the highest values of the distributions with the highest variance, in order to reduce the exponential number of operations in belief propagation. Finally, we propose the implementation of the weighted sum method [33] which approximates the weight matrices in such a way that the number of parameters grows linearly and not exponentially with the number of parents.

In the Results section we compare the response of our model with that of HMAX demonstrating the succesful approximatin of the invariance operation. The dataset is described and used to train and test our model, demonstrating that it is able to account for feedforward categorization, invariant to occlusion, noise, and changes in position and size. Crucially, the inherent properties of Bayesian networks allow us to naturally extend the original feedforward model to include recursive feedback connectivity and account for high-level modulatory effects, such as illusory contour completion and attention, which are also illustrated in the Results section.

In the Discussion section, we provide a comparison with previous models, discuss the biological realism of the model and examine the feedforward and feedbackresults, providing further insights into the model. We conclude by proposing a number of open questions and interesting model extensions for the future.

## Methods

### Multi-stage Hubel-Wiesel Networks and the HMAX Model

This section introduces a common type of object recognition architecture, the multi-stage Hubel-Wiesel network, and describes the HMAX model. The model proposed in this paper attempts to reproduce the structure and functionality of HMAX from a probabilistic perspective and extend it with feedback connectivity.

Modelling visual perception requires building internal representations of the world that are able to capture the relevant information while being invariant to irrelevant variations. The family of models known as Multi-Stage Hubel-Wiesel networks, inspired by biophysiological principles derived from the study of primary visual cortex [34], provide a flexible and trainable hierarchical architecture that can learn selective and invariant features for categorization. Some well known models that belong to this family are Fukushima’s Multilayer Perceptron or Neocognitron [35], LeCun’s ConvNets [30], [36] and Poggio’s HMAX model [31], [37]. The common factor of this set of models is the use of two operations implemented in alternating stages: 1) selectivity, a template-matching or convolutional operation using a set of prototypes (or filter bank), inspired by V1 simple cells; and 2) invariance, a pooling and subsampling operation, inspired by V1 complex cells.

The HMAX model is a well-known model which, since its first publication in 1999 [31], has been further developed and improved in several subsequent versions [37]–[40]. The main difference between HMAX and other multi-stage Hubel-Wiesel architectures is that it has focused on reproducing anatomical, physiological [37] and psychophysical [39] properties of the ventral path of the visual system, comprising areas V1, V2, V4 and IT. For example, the lower-level prototypes in HMAX are not obtained through unsupervised learning as in ConvNets but are hard-wired Gabor filters with physiologically realistic parameters. The model is grounded on widely accepted neurophysiological principles, such as a hierarchical increase in receptive field size and complexity, and shows high level responses that are consistent with our current knowledge of extrastriate cortex functionality. These responses reproduce V4 shape selectivity distributions [41] and predict human performance during a rapid categorization task [39]. Hence, HMAX has been described as the *standard model* [40] and has been employed as the base model to simulate other phenomena such as attention [42], biological motion [43] and learning using spike-time dependent plasticity (STDP) [44]. For these same reasons we have chosen HMAX as our base model, and therefore provide some details of its structure and functionality before describing our proposal.

The version of HMAX we will focus on [37] comprises three different levels representing V1, V2/V4 and IT, which are each subdivided into two layers, simple and complex. Two operations are performed in alternating layers of the hierarchy: the invariance operation, which occurs between layers of the same level (e.g. from S1 to C1); and the selectivity operation implemented between layers of different levels (e.g. from C1 to S2). Each unit in the model receives input from a subset or pool of afferent units in the layer below. For this reason the operations are sometimes denoted as pooling operations, where the pooling size refers to the number of afferent units (similar to the receptive field size).

Invariance is implemented by applying the *max-pooling* function over a set of afferent units selective to the same feature but with slightly different positions and sizes. If any of the afferent simple units within the complex unit’s spatial pooling range is activated, then the complex unit will also emit an equivalent response. If several afferent simple units are active, the response of a complex unit will be equivalent to the response of the afferent simple unit with the highest value. This means complex units achieve a certain degree of invariance to spatial translation and scale.

Selectivity is generated by a template-matching operation over a set of afferents tuned to different features, implemented as a Radial Basis Function network [45]. First, a dictionary of features or prototypes is learned. Each prototype represents a specific response configuration of the afferent complex units from the level below, feeding into the simple unit in the level above. Each simple unit is then tuned to a specific feature of the dictionary, eliciting the maximum response when the input stimuli in the spatial region covered by the unit matches the learned feature. The response is determined by a Gaussian tuning function which provides a similarity measure between the input and the prototype.

Learning in the model takes place at the top level in a supervised way, while at the intermediate levels the feature prototypes are learned in an unsupervised manner. The model implements developmental-like learning, such that units store the synaptic weights of the current pattern of activity from its afferent inputs, in response to the part of image that falls within its receptive field. It simulates the temporal variation in the input images (motion) during learning by moving the RF of a single unit across the whole input image and then generalizing the selectivity features learned to all the units in that layer (*weight sharing*).

For readers who are not familiar with the HMAX model, a more detailed description as well as the equations for each of the layers is included in Text S1.

Taken as a whole the HMAX model provides useful insights into how the selectivity and invariance properties observed along the ventral path can be gradually built. Howver, the model also has several serious limitations. Firstly, learning occurs offline during an initial training stage, and assumes a set of hard-wired features in the lowest level (S1). Secondly, at present the model only provides a static account of the recognition process, i.e. each unit produces a single response for a given input image. This clearly doesn’t capture the complexity and dynamics of neural computations in cortex, and omits challenging aspects, such as the temporal evolution of responses and the interplay between excitation and inhibition to achieve stability. Thirdly, the framework relies entirely on a feedforward architecture, ignoring many connections which are known to exist along the visual pathways. Both long-range horizontal and feedback connections are likely to play an important role in modulating and integrating information across cortical regions [2], [3]. To what degree these are involved in early stages of immediate object recognition is still an open question [46], [47]. Nonetheless the the lack of feedback connectivity has been identified by the authors as one of the main limitations of their model [37].

Our proposal contributes to mitigating the second and third limitations by completely reformulating a simplified version of the HMAX model under a probabilistic framework that includes the temporal dimension and feedback connectivity.

### Bayesian Networks and Belief Propagation

This section provides a definition of Bayesian networks and introduces the equations of the loopy belief propagation algorithm. Several modifications will be introduced later on to some of these equations in order to facilitate their implementation within the large-scale proposed model.

A Bayesian network is a specific type of graphical model called a *directed acyclic graph*, where each node in the network represents a random variable, and arrows establish a causal dependency between nodes. Therefore, each arrow represents a conditional probability distribution 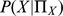 which relates node 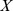 to its parents 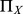. Crucially, the network is defined such that the probability of a node 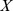 being in a particular state depends only on the state of its parents, 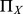. Consequently, a Bayesian network of 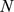 random variables 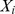 defines a joint probability distribution which can be factorized as follows,
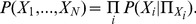
(1)


More formally, a Bayesian network is a pair 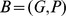, where.

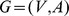
 is an acyclic directed graph with 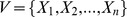, a set of nodes (vertices); and 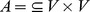, a set of arcs defined over the nodes;

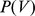
, a joint probability distribution over 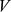, given by Equation (1).

Given the structure of the network and the conditional probabilities defining the joint probability distribution (Equation (1)), it is possible to analytically compute the marginal probability of each node, in terms of sums over all the possible states of all other nodes in the system i.e. using marginalization.

However, this computation is impractical, specially for large networks, as the number of terms in the sums grows exponentially with the number of variables. Furthermore, there are many common intermediate terms in the expressions for the different marginal probabilities, which implies a high redundancy and thus low efficiency in the calculations. Additionally, when new evidence arrives into the network, the effects of the *observed* node modify the marginal probabilities of all other nodes, requiring the whole marginalization process to be repeated for each variable.

Belief propagation is a message-passing algorithm that manages to perform inference in a singly-connected Bayesian network in a way that grows only linearly with the number of nodes, as it exploits the common intermediate terms that appear in the calculations. In belief propagation the effects of the observation are propagated throughout the network by passing messages between nodes. The final belief, or posterior probability, is computed locally at each node by combining all incoming messages, i.e. evidence from higher and lower levels.

Note for nodes without parents (root nodes), the conditional probability of 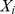 is equal to its prior probability, i.e. 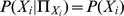. Thus, defining the whole structure of a Bayesian network requires specification of the conditional probability distribution of each node with parents, 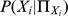, plus the prior probability distributions of all root nodes, 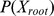.

In Bayesian networks with loops the original belief propagation algorithm is no longer valid and approximate methods have to be employed. One such method is loopy belief propagation, which naively implements the original belief propagation algorithm leading to messages circulating in the network indefinitely. However, for pyramidal networks, such as the ones considered here, the method has been empirically demonstrated to obtain good approximations to the exact beliefs, once the approximate beliefs have converged after several iterations [48]. See [49] for a more detailed exploration of loopy belief propagation and its relation to Bethe free energy minimization.

**Figure 1:**
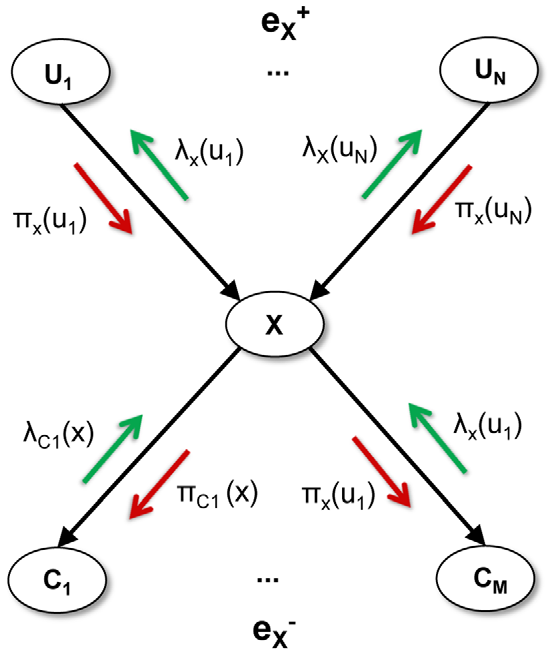
Message passing in belief propagation in a Bayesian network. Node 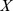 receives all bottom-up messages 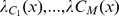 from its children, and all top-down messages 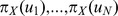 from its parents. The belief can then be calculated by combining all bottom-up evidence 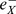 and top-down evidence 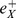. Node 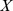 generates outgoing messages 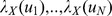 for its parent nodes, and messages 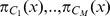 for its child nodes.

Below we describe the computations performed locally by a node in the generic section of a hierarchical Bayesian network represented in Figure 1. Note that the equations include the temporal dimension because they are capturing the loopy belief propagation algorithm, which requires several iterations to converge. Given a node 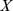 with parent nodes 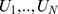, and a set of child nodes 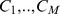, the loopy belief propagation operations for each node can be described in three steps:

1. Node 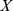 receives all bottom-up messages 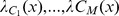 from its children, and all top-down messages 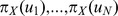 from its parents.
2. Given the fixed conditional probability distribution 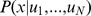 that relates node 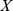 to its immediate parents 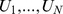, node 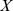 can calculate its belief as
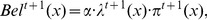
(2)

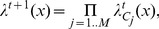
(3)

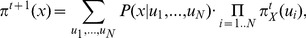
(4)

where 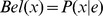, represents the probability of node 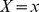 given some evidence 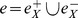, and is usually referred to as the posterior probability or Belief; 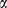 represents a normalization constant; 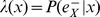 represents the diagnostic or *retrospective* support that the assertion 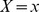 receives from 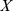
’s descendant, and is usually referred to as the likelihood function; 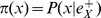, represents the causal or *predictive* support that the assertion 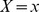 receives from all non-descendants of 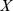, via 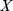
’s parents, and is usually referred to as the prior function;

1. Node 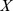 generates outgoing messages 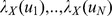 for its parent nodes, and messages 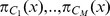 for its child nodes, given by the following equations:
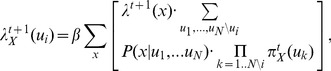
(5)

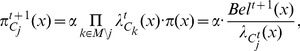
(6)

Where 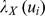 represents the bottom-up message that node 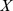 receives from node 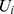; and 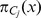 represents the top-down message that node 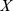 sends to node 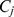.

Note the 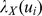 message can be sent to node 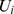 as soon as messages from all other nodes, except node 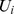, have been received. Analogously 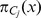 can be sent as soon as all messages, except that arriving from node 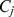, have been received.

**Figure 2:**
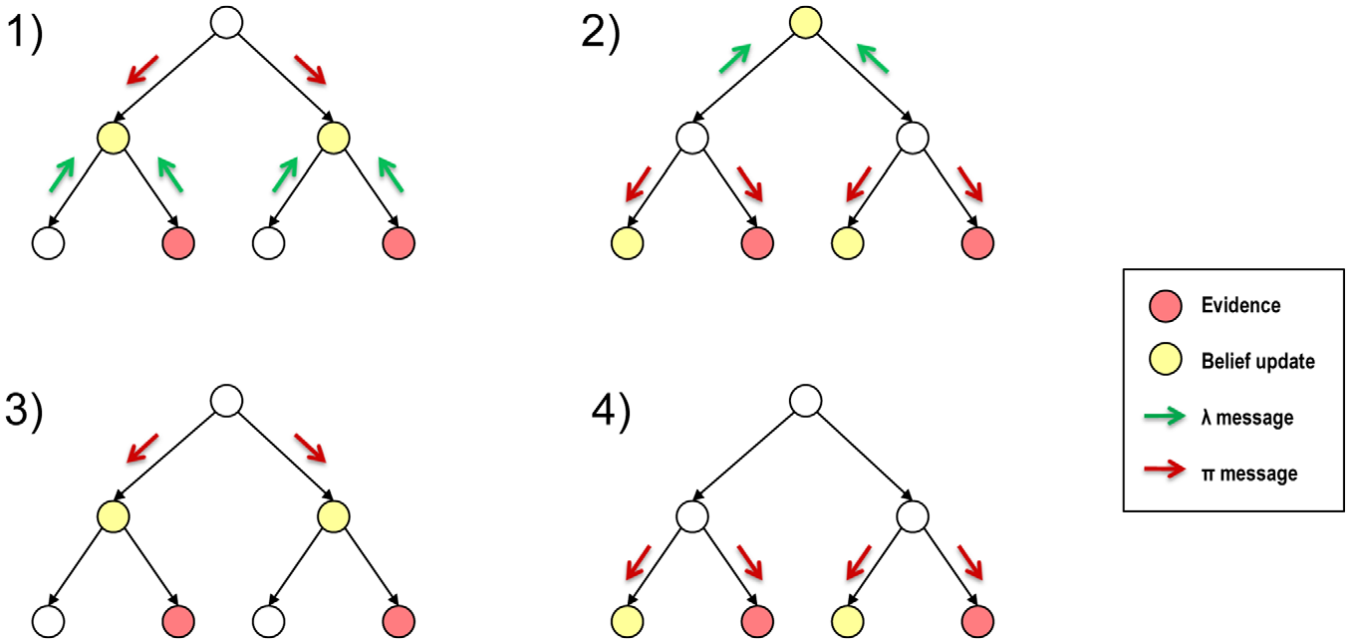
Example of belief propagation in a tree-structured network. The network has three levels organized in a tree structure. In the first step evidence propagates from two of the child nodes in the lower level, leading to the update of the belief in the intermediate nodes. In the second step, the belief at the top level is updated, together with the belief at the lower-lever child nodes that hadn’t been instantiated. The crucial process occurs in step three when a message is sent downward from the top node. The top node receives messages from the two intermediate child nodes (the left and the right branches of the tree), and therefore it must generate a top-down message for each node conveying the evidence collected from the other node. In other words the evidence from the left branch must be propagated to the nodes in the right branch and vice versa. This is shown in steps three and four.

Figure 2) illustrates belief propagation by showing how messages and beliefs evolve in a simple tree-structured network with three levels. In the first step, evidence propagates from two of the child nodes in the lower level, leading to the update of the belief in the intermediate nodes. In the second step, the belief at the top level is updated, together with the belief of the lower-lever child nodes that hadn’t been instantiated. The crucial process occurs in step three when a message is sent downward from the top node. The top node receives messages from the two intermediate child nodes (the left and the right branches of the tree), and therefore it must generate a top-down message for each node conveying the evidence collected from the other node. In other words the evidence from the left branch must be propagated to the nodes in the right branch and vice versa. This is depicted graphically in steps three and four.

### Bayesian Network with HMAX-like Architecture

This section provides a description of how to generate a Bayesian network with a structure similar to that of HMAX. It also provides an overview of our proposed model including its architecture, parameters, input and output.

**Figure 3:**
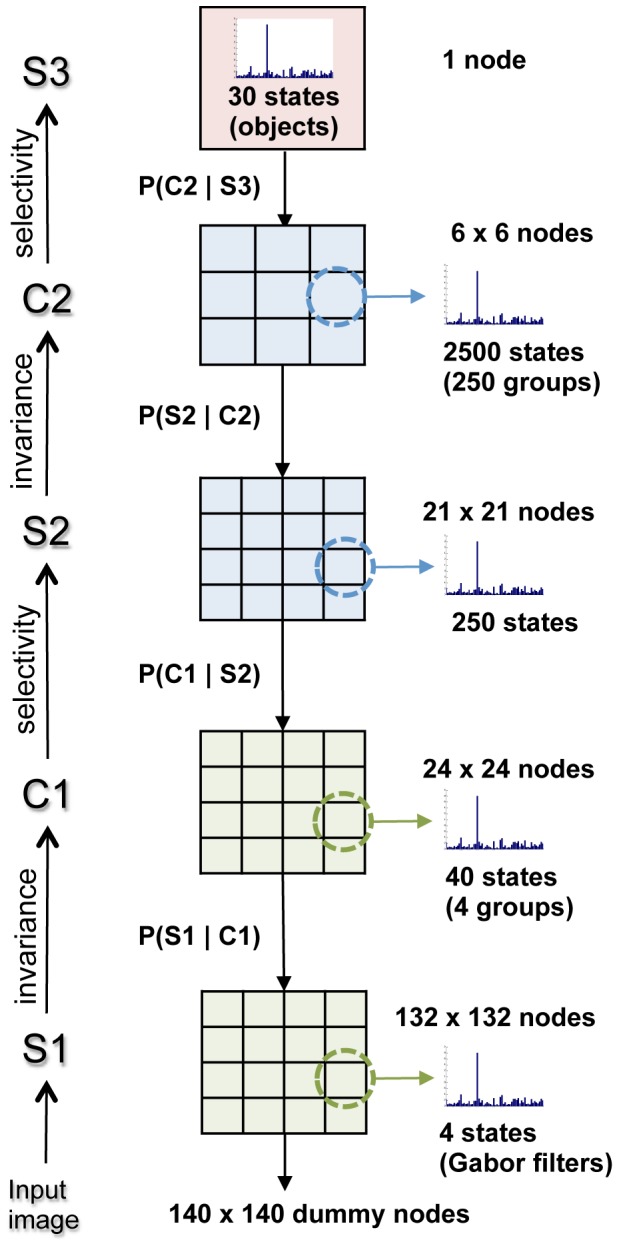
Schematic representation of the HMAX-like Bayesian network. The 5-layer Bayesian tries to replicate the structure and functionality of a simplified version of the HMAX model [37]. The probability distribution of each Bayesian node (grid square) represents the sum-normalized response of HMAX units at that location and layer, where the states of the node represent the different features (e.g. four Gabor filters). The conditional probability tables linking the nodes of different layers serve to approximate the HMAX selectivity and invariance operation (see text for details). The number of nodes per layer and the number of states per node is indicated beside each layer. The downward arrows between the layers (square grids) indicate the causal dependencies in the Bayesian network, e.g. C1 nodes are the parents of S1 nodes and the children of S2 nodes. The specific connectivity patterns between nodes are shown in Table 1.

Our proposed model consists of a Bayesian network that reproduces the structure of a specific HMAX version with five layers [37]. Although Bayesian networks (directed acyclic graphs) can also be formulated as undirected graphical models, such as factor graphs or Markov random fields, the directionality of Bayesian networks fits better with the generative modelling approach proposed to model vision [5]. The specific parameters of this implementation, which were used to obtain the feedforward categorization and feedback modulation results in this paper, are shown in Table 1 and illustrated in Figure 3.

**Table 1:** Model Parameters.

Name	Value	Description
*N_S_*_1_	9×9	RF size of dummy nodes (Gabor filters)
*K_S_*_1_	4	Number of states (features) in S1 nodes = Gabor filter orientations, (0°; 45°; 90°; 135°)
*N_C_*_1_	10×10	RF size of C1 nodes (number of S1 nodes pooled)
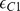	5	Step between C1 nodes (in number of S1 nodes) - sets the C1 downsampling factor
*K_C_*_1_	40	Number of states in C1 nodes
*K_C_*_1*group*_	10	Number of states per *group* in C1 nodes. The number of C1 *groups* = 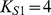 , such that 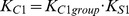 .
*N_S_*_2_	4×4	RF size of S2 nodes (number of C1 nodes pooled)
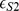	1	Step between S2 nodes (in number of C1 nodes) - sets the S2 downsampling factor
*K_S_*_2_	250	Number of states (features) in S2 nodes
*N_C_*_2_	6×6	RF size of C2 nodes (number of S2 nodes pooled)
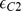	3	Step between C2 nodes (in number of S2 nodes) - sets the C2 downsampling factor
*K_C_*_2_	2500	Number of states in C2 nodes
*K_C_*_2*group*_	10	Number of states per *group* in C2 nodes. The number of C2 *groups* = 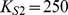 , such that 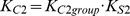 .
*K*_S3_	30	Number of states in the S3 node = number of objects or categories
*N_S_*_3_	6×6	RF size of S3 node (number of C2 nodes pooled)

Parameters of the HMAX-like Bayesian network. Note some of the results may be shown as a function of different values of these parameters.

Note that in this implementation of the network we have omitted the scale bands (i.e. features maps obtained for different pooling sizes) of layers S1, C1 and S2. Results from a previous implementation of the Bayesian network, which included all the scale bands [50], showed that the higher scale bands, with large pooling size and low resolution, did not provide a significant improvement during feedforward categorization of the current dataset (object silhouettes of 140×140 pixels). Additionally, the feedback effects were more diffuse and the simulation time increased drastically.

The steps required to define a Bayesian network with an HMAX-like structure are are as follows:

1. Each node of the Bayesian network represents a specific location and layer of the HMAX model.
2. The discrete states of each node of the Bayesian network represent the different features coded at that location and layer of the HMAX model. For example each Bayesian node at layer S1 will have 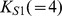 states, representing the four different Gabor filter orientations of HMAX.
3. The discrete probability distribution over the 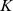 states of each Bayesian node comprises the sum-normalized responses of the 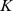 HMAX units coding the different features at that location and layer.
4. The conditional probability tables (CPTs) that link each node of the Bayesian network with its parent nodes in the layer above, represent the prototype weights used to implement selectivity in the HMAX model. Additionally, the CPTs are used to approximate the *max-pooling* (invariance) operation between simple and complex layers of the HMAX model. Learning the appropriate CPT parameters allows the model to approximate the HMAX functionality using loopy belief propagation.

Figure 3 shows a schematic representation of the proposed Bayesian network model. The input image is pre-processed with a battery of Gabor filters of size 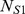 i.e. at 4 different orientations. Each of the filters is applied at every location of the image. The filtered responses, normalized over the four orientations at each location, are used as the output 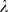 messages of a set of dummy nodes that feed onto the S1 nodes. Dummy nodes do not encode a variable or have a belief, they simply generate 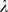 messages for the parent nodes. The rest of the layers, from S1 to S3 are implemented following the methodology for simple and complex layers described in the following section. The top layer employs supervised learning where the weights (prototypes) for each of the states corresponds to the output of the C2 layer for each of the object categories. Thus, layer S3 contains a single node with a probability distribution over the learned object categories, which can be used to to evaluate the categorization performance of the model.

Notably, every node in the Bayesian network has an identical internal structure implementing the loopy belief propagation algorithm. The following section describes, using a toy-example, how to approximate the invariance and selectivity operations using this algorithm, whereas the section afterwards describes the learning methods required to to obtain this functionality. The last two sections within the Methods detail several approximations that simplify the algorithm and allow it to run in the large-scale Bayesian network proposed.

### Approximating the Invariance and Selectivity Operations Using Belief Propagation (a Toy Example)

In this section we provide a comprehesive description of how our proposed model works, using a toy-example. We start with a general overview of the toy-model and then include the particularized equations and a numeric example to illustrate its functionality.

**Figure 4:**
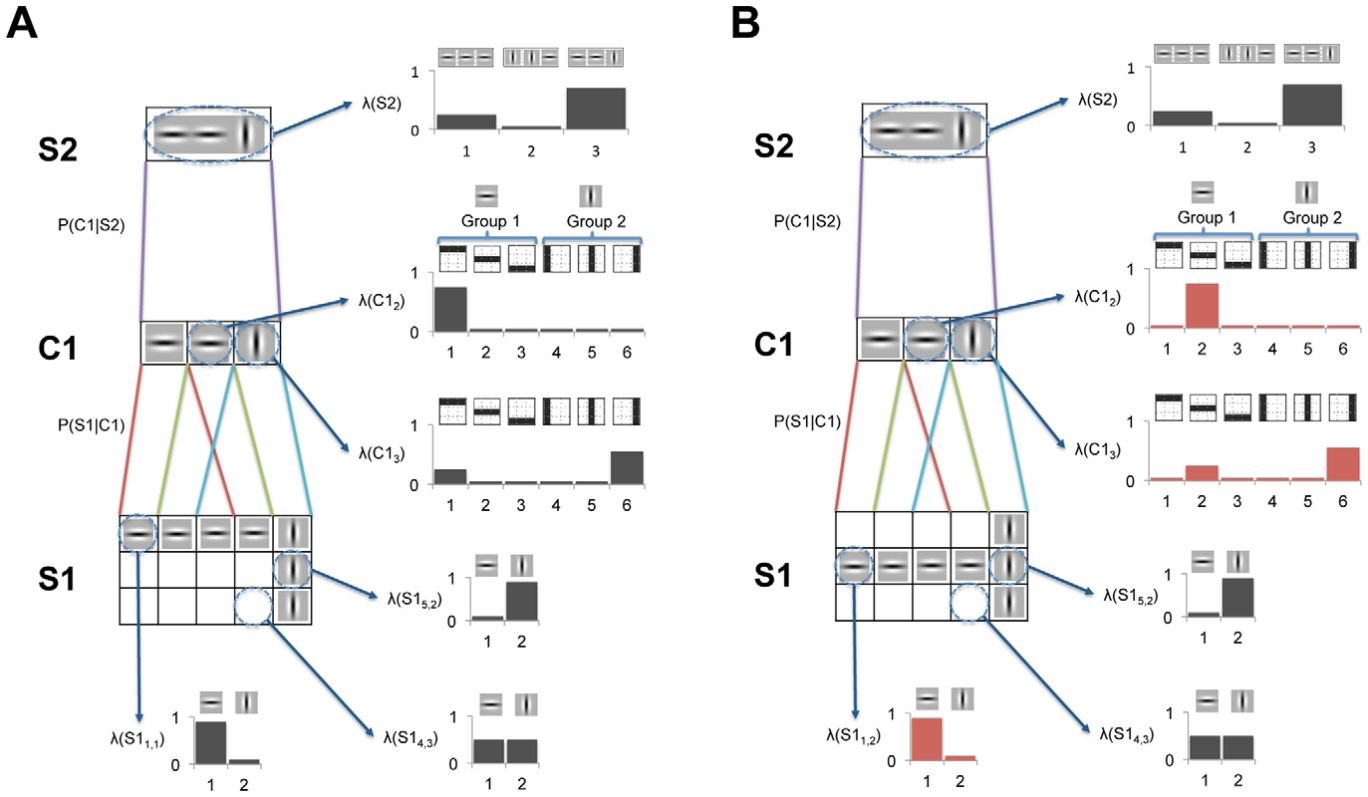
Toy-example of Bayesian network that approximates the selectivity and invariance operations using belief propagation. Each square of the grid represents a Bayesian node, such that there are 15 S1 nodes, 3 C1 nodes and 1 S2 node. Each C1 node has 3×3 child nodes in layer S1 delimited by the red, green and light blue lines; whereas the S2 node has 3 child nodes in layer C1 (purple lines). Several nodes are circled and have a blue arrow pointing to the probability distribution over the states of that node. A small picture of the feature or prototype associated with each state is shown above it. The feature (e.g. horizontal Gabor filter) corresponding to the most probable state of each node is represented inside the node. Nodes with equiprobable distributions are left empty. C1 states are clustered in *groups*, where each *group* can be interpreted as a position-invariant representation of an S1 state/feature. S2 prototypes are learned as a function of C1 *groups* (CPTs have the same weight for all C1 states within a *group*) which allows to achieve certain position invariance. For the Bayesian network in panel B we assume the input image has been moved slightly downward as compared to the input of panel A. This leads to a set of different S1 and C1 probability distributions in panel B (changes are highlighted in red). However, because the new C1 winner states belong to the same *group* as in panel A, the same messages are sent from C1 to S2 and consequently the S2 probability distribution will also be identical, demonstrating selectivity and invariance in the Bayesian network. See text for details.

We start by defining a toy-example scenario composed of a three layer Bayesian network with an HMAX-like structure, i.e. alternating simple and complex layers (S1, C1 and S2), as shown in Figure 4. Our aim is to approximate the HMAX invariance operation, typically implemented in C1 nodes by *max-pooling* over a subset of S1 nodes, and the selectivity operation, typically implemented in S2 by performing a distance operation (Radial Basis Function) between a subset of C1 nodes and a learned set of prototypes.

Figures 4A and 4B represent the same toy-example Bayesian network with two different inputs. In these figures, each square of the grid represents a Bayesian node, with 15 nodes in layer S1 (labelled according to their X,Y location), 3 nodes in layer C1 and 1 node in layer S2. The coloured lines represent the connectivity or, in Bayesian terms, the causal dependencies between the nodes. For example, the 3×3 S1 nodes on the left, 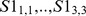, feed onto node 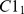 (red lines), which means that 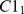 is the parent node of 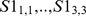. Similarly, 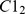 is the parent node of 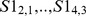 (green lines) and 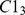 is the parent node of 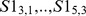 (green lines). Analogously, the top node 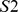 is the parent node of the three C1 nodes, 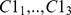 (blue lines).

Several nodes in Figure 4A are circled and have a blue arrow pointing to the probability distribution over the states of that node. A small picture of the feature or prototype associated with each state is shown above it. In this toy-example, the S1 nodes have two states corresponding to the horizontal and vertical Gabor filters, which from now on will be denoted as the *horizontal* and *vertical* states, respectively. The probability of each state will depend on the response of the corresponding Gabor filter to a specific image patch feeding into that node (not shown in the figure). For example, in node 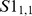 the *horizontal* state exhibits a high probability, indicating it receives input from an image patch with horizontal contours; whereas node 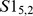 shows a high probability for the *vertical* state indicating it receives input from a region of the image with vertical contours. S1 nodes, such as 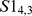, whose input corresponds to blank regions of the image will have a flat probability distribution, indicating all states are equally likely. The feature, horizontal or vertical Gabor filter, corresponding to the most probable state of each node is represented symbolically inside the grid square of that node. Nodes with equiprobable distributions are left empty.

Each C1 node in this toy-example has six states corresponding to different combinations of the states of the 3×3 S1 nodes feeding into it. These combinations can also be understood as the C1 features or prototypes and are encoded in the weight matrix (CPTs) between the S1 and C1 nodes. For example, in Figure 4A, C1 state 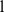 will exhibit a high probability if the *horizontal* state of the top three S1 nodes shows high probability. This is the case of node 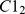, so we can state that 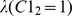 is high because 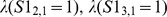 and 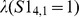 are high. On the other hand, the probability of C1 state 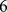 will be high if the probabilities of the *vertical* state of the three rightmost S1 nodes is high. This is the case of node 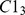, for which we can say that 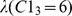 is high because 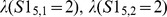 and 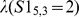 are all high. Interestingly, node 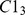 also exhibits a moderate probability for C1 state 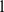, given that two out of the three top S1 nodes have high probabilities in the *horizontal* state.

We now introduce the concept of *groups*, which is one of the key novelties that allows the implementation of the *max-pooling* operation in Bayesian networks. *Groups* are only present in complex, i.e. layers starting with ‘C’ where nodes represent complex cells and implementing the invariance operation. A *group* is defined simply a subset of the states of a node. This subset of states will share a common pattern, for example responding preferentially to three horizontally aligned S1 nodes with high *horizontal* state probabilities. This is the case of C1 *group* 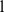, which subsumes states 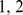 and 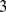. The corresponding *group* 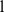 feature or prototype can be understood as a spatially invariant horizontal contour (symbolized as a horizontal Gabor filter). The reason for being spatially invariant is that it is associated with horizontal contours at three different positions, those represented by C1 states 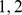 and 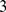. Analogously, C1 *group* 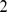 comprises states 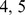 and 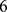, and its associated feature can be interpreted as a spatially invariant vertical contour (symbolized as a vertical Gabor filter). Importantly, the number of C1 *groups* (C1 invariant features) is equal to the number of S1 states (S1 features). The invariant feature associated with the *group* with the highest probability (summing the probabilities of its states) is represented symbolically inside the grid square of each C1 node. For example, node 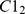 contains a horizontal Gabor filter symbol indicating that *group* 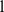, associated with a spatially invariant horizontal contour, has the highest sum of probabilities.

The top layer S2 node has three states, each corresponding to different combinations of the states of its afferent C1 nodes. Again, these combinations can be interpreted as the S2 features or prototypes and are encoded in the CPTs between C1 and S2 nodes. When learning these CPTs we make a key assumption, namely, that C1 states belonging to the same *group* will have the same weight. This will become clearer below, once the equations and CPTs of this toy-model are described, but, intuitively, this means that the S2 states can be defined in terms of the C1 *groups* of the three afferent C1 nodes. For this reason, the symbolic representation of each of the three S2 states is shown as combinations of three C1 *group* features. For example, C2 state 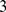 will show a high probability when the *groups* 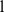 (invariant horizontal contour) of nodes 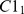 and 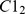, and *group* 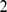 (invariant vertical contour) of node 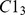 have high probabilities. The S2 state with highest probability is represented symbolically inside the rectangle representing node 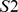.

A crucial aspect to clarify here is that the concept of *groups* is external to the Bayesian network and does not modify in any sense the definition of nodes or states. As will be described below, *groups* simply provide a convenient way of clustering states during the learning phase, but the underlying Bayesian network remains conventional in every sense. In fact, once the CPTs are learned, inference can be performed in the network ignoring the concept of *groups*.

After providing a general description of the toy-example we now provide its mathematical parameters and equations. These follow the same nomenclature as the parameters and equations that describe the full large-scale model (see Table 1 and Figure 3). This will help the reader extrapolate the ideas conveyed by the toy-example to the real model.

The parameter values for the toy example are: 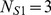
×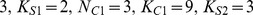, where 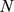 represents the pooling sizes and 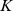 the number of states. Additionally, the parameter 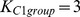 is defined, meaning that each *group* is composed of 3 C1 states. This satisfies the equation 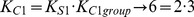, i.e. the number of C1 states is equal to the number of S1 states (the number of *groups*) times the number of C1 states per *group*.

The S1 nodes of this toy-example Bayesian network have multiple parents, i.e. the receptive fields of C1 nodes overlap, exemplifying the loops that are present in the full large-scale Bayesian network. However, for the sake of clarity, here we provide the equations of a singly-connected network (one parent per node). The specific equations to deal with multiple parents and loops will be discussed in a subsequent section. Given that we are currently focusing on the feedforward operations of the model, only Equations (5) and (3), which refer to the bottom-up messages in belief propagation, are particularized for the toy-example:
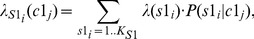
(7)

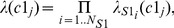
(8)

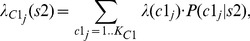
(9)

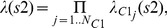
(10)where low-case letters denote specific states of a node. If one compares the set of Equations (7) and (8) with (9) and (10), it may be striking that they have the same form despite implementing different functionalities at different layers. However, this is not surprising given that they both correspond to the belief propagation algorithm, which, by definition, requires that every node carries out the same operations. This conundrum is resolved by realizing that it is the CPTs that determine effective connectivity of the network and, consequently, the functionality of the algorithm at each layer. In our case, Equations (7) and (8) implement a necessary pre-processing step required to approximate the *max-pooling* operation, Equation (9) approximates the *max-pooling* operation and Equations (9) and (10) approximate the selectivity operation.

**Figure 5:**
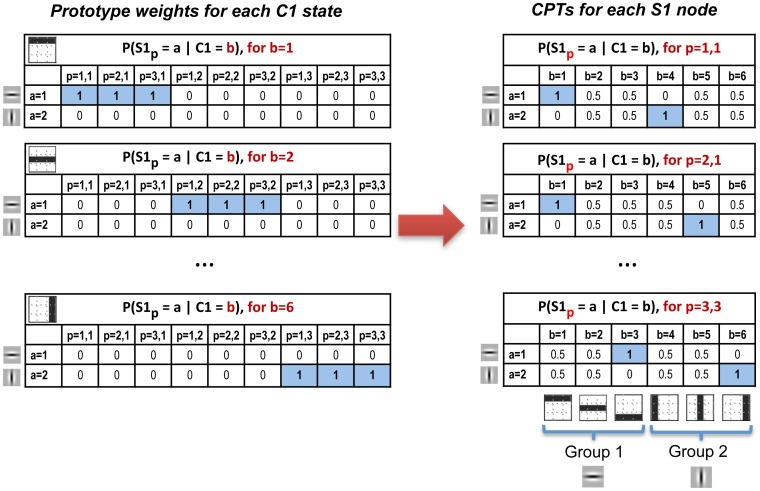
Prototype weight matrices and CPTs between S1 and C1 nodes in the toy-example. Let 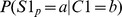 be the CPT between 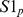 and 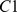 for S1 state 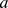 and C1 state 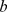. The left column shows the HMAX-like prototype weights where an individual table is learned for each of the 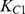 C1 states (prototype), 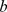, as function of the afferent 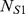 x 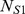 S1 nodes, 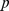, and the 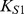 S1 states, 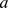. However, the CPTs of a Bayesian network are defined as a function of the 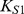 child states, 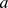, and the 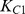 parent states, 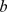, for each of the 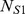 x 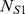 child nodes, 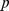. Therefore, once the weight matrices are generated for each C1 state, they need to be converted to the corresponding CPTs of each S1 node (right column). To conform to probability rules each column of the CPT, the distribution over the child node states, is sum-normalized to one (empty columns are converted to equiprobable distributions). C1 states are clustered in *groups* to help approximate the invariance operation (see text for details). The feature/prototype symbols associated with each state are included in the figure.

**Figure 6:**
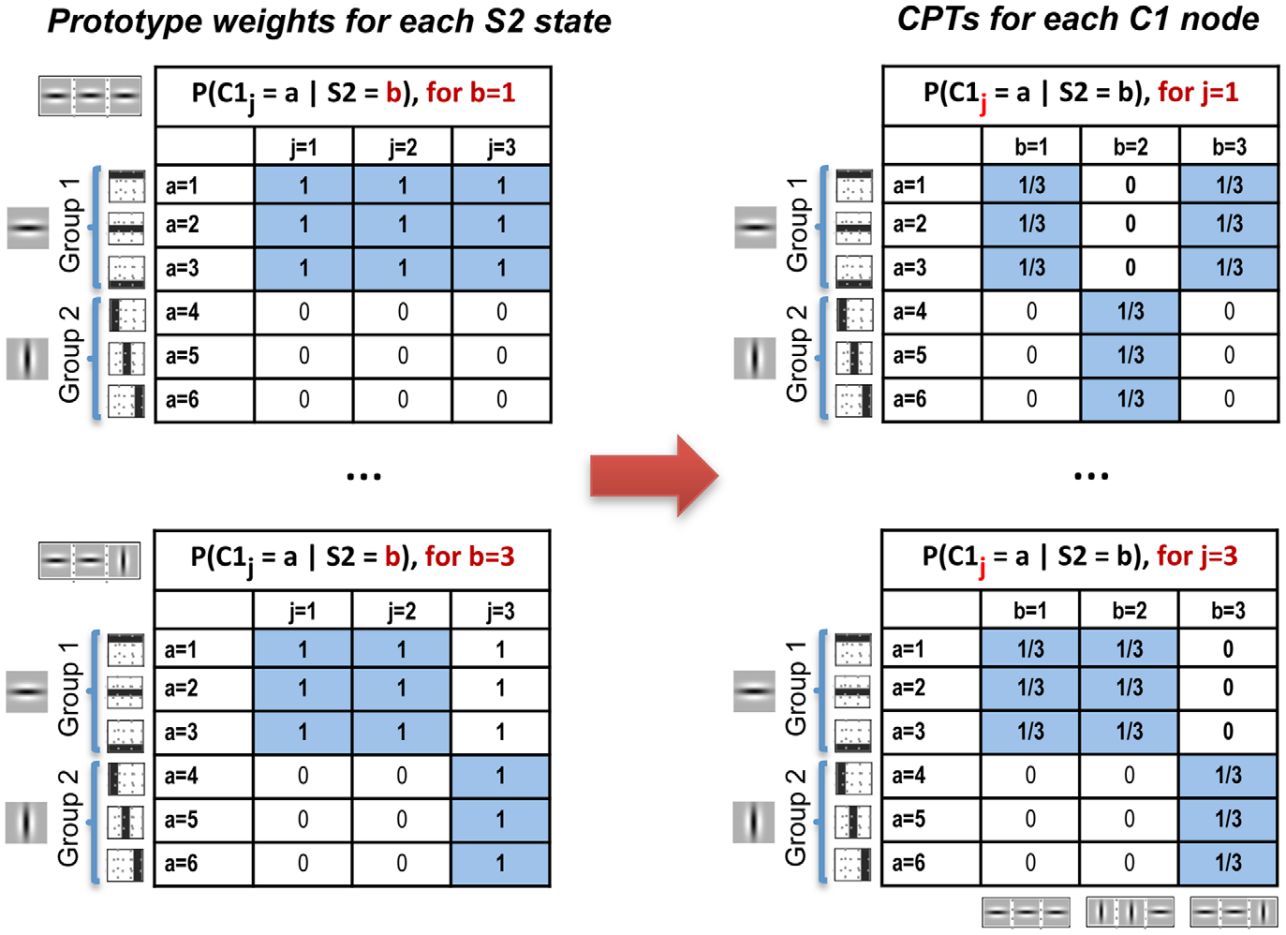
Prototype weight matrices and CPTs between C1 and S2 nodes in the toy-example. Let 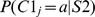 be the CPT between 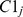 and 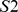 for C1 state 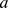 and S2 state 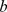. The left column shows the HMAX-like prototype weights where an individual table is learned for each of the 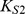 S2 states (prototype), 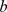, as function of the afferent 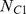 C1 nodes, 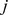, and the 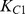 S1 states, 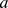. However, the CPTs of a Bayesian network are defined as a function of the 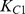 child states, 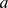, and the 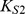 parent states, 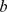, for each of the 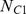 child nodes, 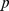. Therefore, once the weight matrices are generated for each S2 state, they need to be converted to the corresponding CPTs of each S1 node (right column). To conform to probability rules each column of the CPT, the distribution over the child node states, is sum-normalized to one (empty columns are converted to equiprobable distributions). Importantly, S2 prototypes are actually learned as a function of C1 *groups*, such that the weights of C1 states belonging the same *group* are equal, which helps to approximate the invariance operation (see text for details). The feature/prototype symbols associated with each state are included in the figure.

The CPTs 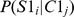 and 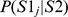 can be derived from Figures 5 and 6. It is important to note that the prototype weights for each C1 state are learned as a function of the 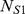
×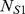 afferent S1 nodes and the 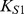 S1 states per node (left column of Figures 5). This yields a weight matrix for each C1 state. However, the CPTs of a Bayesian network are defined as a function of the child and the parent states, 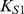 and 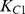, for each of the child nodes, 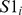. Therefore, once the weight matrices are generated for each C1 state, they need to be converted to the corresponding CPTs of each S1 node, 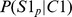 (right column of Figure 5). To conform to probability rules each column of the CPT, the distribution over the child node states, is sum-normalized to one (empty columns are converted to flat distributions). The same applies to 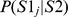, shown in Figure 6.

Although learning will be described in more detail in the following section, we point out here that *weight sharing* is used in the model. This means that the prototype weights between 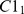 and its afferent S1 nodes, 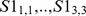, are the same as those between 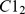 and its afferent S1 nodes, 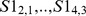. In turn this means that, assuming the network was singly-connected, the following equation holds: 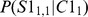  =  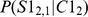  =  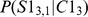 and so on. Consequently, it is possible to calculate 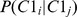 for any 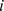 and 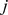, given the generic CPTs 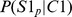 (Figure 5), where 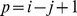.

We will now describe in more detail each of the steps using a numerical example for the toy-example. Equation (7) generates the message sent from each S1 node to its parent C1 node. Each message, 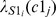, conveys a probability distribution over the states of C1 based on the evidence of the S1 node, . Let S1 nodes with a horizontal Gabor filter symbol have 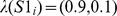; and those in blank have 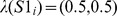, as illustrated in Figure 4A. Given the CPTs in Figure 5, the message sent to 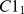 from 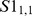 is 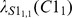 = 0.9⋅(1,0.5,0.5,0.5,0.5,0.5)+ 0.1⋅(0,0.5,0.50.5,1,0.5,0.5) = (0.9,0.5,0.5,0.1,0.5,05), indicating that the most probable state of 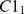 according to 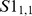 is state 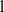, which is what one would intuitively expect when looking at the feature symbols. The same message is conveyed to node 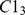 by nodes 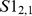 and 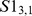. However, the messages sent to 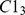 from 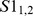, 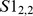, 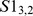, 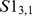, 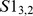, 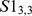 will have a flat distribution, indicating all C1 states are equally probable.

The messages from the 3×3 afferent S1 nodes of 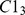 are then multiplied together (Equation (8)), yielding (after normalization) 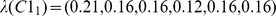. This indicates that the most probable state of 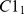 given the evidence provided by all of its child nodes, is state 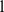 (note that for clarity the distributions in Figure 4 are not shown to scale). This demonstrates that by learning the appropriate CPTs it is possible to associate each C1 state with a particular combination of S1 states and nodes. As previously described, C1 states are divided into *groups*, where each *group* is associated with a particular S1 state. The C1 states within a *group* correspond to common patterns, observed during training, of the afferent S1 nodes with high probabilities for that state. For example, C1 *group* 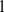 captures three typical arrangements of S1 nodes that contain high probabilities for *horizontal* state: three adjacent S1 nodes at the top row (C1 state 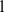), middle row (C1 state 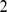) and bottom row (C1 state 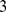).

Equation (9) generates the message between each C1 node and its parent S2 node. Let 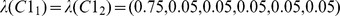 and 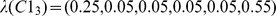 (we choose these more extreme and uniform values to make the example clearer). The message sent from 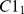 to 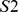 is 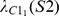 = 0.75⋅(1/3,0,1/3)+0.05⋅(1/3,0,1/3)+0.05⋅(1/3,0,1/3)+0.05⋅(0,1/3,0)+0.05⋅(0,1/3,0)+0.05⋅(0,1/3,0) = (0.46,0.08,0.46), which indicates that according to the evidence from node 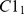 the most probable S2 states are 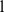 and 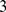, consistent with the feature symbols. The important point to highlight here is that the CPT weights of C1 states belonging the same *group* are equal (right column of Figure 6). Consequently, the same message would be sent to 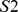 from 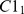 if either state 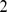 or state 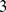, instead of state 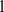, had a high probability. This is illustrated in Figure 4B (with changes highlighted in red), where we assume the input image has changed (moved slightly downward) leading to a different set of S1 probability distributions. The C1 probability distributions, 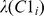, have also changed but, because the new winner states belongs to the same *groups* as before, the messages sent to S2 will be the same, leading to the same S2 probability distribution. This demonstrates position translation invariance in the Bayesian network.

We have argued that this invariance is achieved by approximating the *max-pooling* operation using (9). However, how well this operation is approximated depends on the number of C1 states per *group* that have high probabilities, where the ideal case would be to have a single one per *group*. To try to minimize the number of simultaneous highly probable C1 states, the weights of C1 states within a *group* are learned using k-means clustering, which, as described in the following section, tries to minimize the similarities between the different C1 prototypes. However, this doesn’t guarantee that several C1 states in a group won’t have high probabilities. If that is the case, (9) can still be interpreted as achieving invariance by approximating a more relaxed or alternative version of *max-pooling*, such as *soft-max* or *average-pooling*, which have also been employed in HMAX [38] and similar models such as ConvNets [30]. In all cases, belief propagation can only be considered to an approximation to any of these pooling operation because it is not taking into account all the afferent responses (i.e. all states from all nodes) but only the most common combinations of these. The validity of this approximation will depend on the ability of the learning algorithms to extract combinations that capture well the statistical properties of the image dataset and allow for generalization.

The last stage is the approximation of selectivity, which basically consists of finding the distance between the input and a set of prototypes. We have already described how 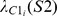 (Equation (9)), conveys the probability distribution over the S2 states based on the evidence from each C1 node. Here, the probability values can be interpreted as the distance between the input and the set of prototypes, which represented by the S2 states. Following the numeric example from the toy-example we have 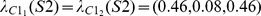 and 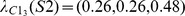. In other words, messages from 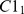 and 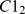 suggest that S2 states 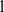 and 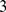 are the most probable, whereas the message from 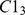 suggests that S2 state 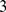 is the most probable. To obtain the final feedforward probability distribution over S2 states, the messages from all child nodes are multiplied together (Equation (10)), yielding 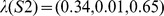 (not to scale in Figure 4), indicating the most probable S2 state is 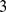. This can be interpreted as the S2 prototype corresponding to state 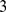 being the one that better captures (has minimum distance to) the input to the network. The same result is obtained for the network with slightly different input shown in Figure 4B, given that S2 prototypes are built from a set of C1 position invariant features. Belief propagation still only constitutes an approximation to selectivity because the selectivity is usually implemented as a weighted sum whereas here we employ the product of a set of weighted sums. However, the latter method has also been used as a selectivity operation in HMAX-like models such as HTM [13].

### Learning

This section describes the algorithms required to learn the CPTs of the Bayesian network nodes. Two algorithms are described, one for nodes implementing the invariance operation and the other for nodes implementing the selectivity operation.

Training in the networks occurs in a bottom-up discriminative manner, one layer at a time, by freezing the weights of the layer below and using its activation to learn the next layer. In other words, learning is layerwise greedy, as in deep learning models [13], [32]. Because the CPTs have not yet been learned it is not possible to compute the top-down prior component, 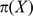, of the belief. A possible solution is to assume a set of initial parameters, perform inference and refine the parameters in several iterations of inference and learning (the Expectation-Maximization approach). However, this method is extremely demanding and infeasible in relatively large networks. Therefore, to deal with the effect of top-down 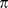 messages during training we assume each node has no parents when computing its belief and has a single parent during the generation of the output 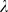 message. This eliminates the feedback component making the belief at each node equivalent to the bottom-up likelihood function, 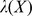.

In order to reduce the memory required to store the network parameters and speed up the belief propagation algorithm, the weights are shared amongst the nodes of each layer, i.e. the CPT between each parent node and its child nodes nodes is identical for all nodes in the same layer. This *weight sharing* technique, inspired by developmental-like learning of V1 and V2 neurons [37], simulates the temporal variation in the input images by moving the receptive field across all locations and then using the same selectivity weights for all nodes of the layer.

Importantly, we note that there are no weights between the Dummy nodes and the S1 nodes given that we are replicating the HMAX model, whose S1 features are hard-wired to Gabor filters. Dummy nodes are the nodes that interface the input image with layer S1, and are called “dummy” because they don’t store a Belief but simply send a 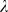 message to each S1 node. Each 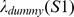 message has four values corresponding to the Gabor filter responses at four orientations applied over the region of the image associated with the S1 node (see Text S1 for the Gabor filter equation and parameters). Consequently, given that each S1 node receives a single message, we can write 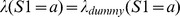.

We now describe how to learn the weights between the simple and complex layers (e.g. S1 and C1), which help to approximate the invariance operation. Note that, unlike in the toy-example, in the large-scale model there are four S1 states representing Gabor filters at four different orientations. Learning the CPTs 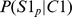 requires finding for each S1 state, the 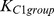 most common patterns of the 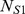
×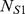 S1 nodes feeding into its parent C1 node. Furthermore, we need to ensure that the different patterns learned are as dissimilar as possible in order to minimize the number of active C1 states at a time in each *group*. To do this we apply k-means clustering over all the different afferent patches of S1 nodes obtained from the training dataset. This is done independently for each S1 state and fixing the number of clusters to 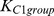. Given that k-means is especially sensitive to initial starting conditions, we implement a procedure for computing a refined starting condition that leads to improved solutions [51].

Let 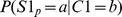 be the CPT between 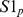 and 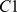 for S1 state 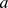 and C1 state 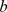. As described in the previous section, initially an individual table is learned for each C1 state (prototype) 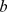 as function of the afferent S1 nodes 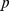 and S1 states 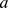. However, these weights need to be converted to the CPTs of the Bayesian network, which are defined as an individual table for each S1 node 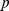 as a function of the child states 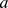 and the parent states 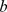. To conform to probability rules each column of the CPT, the distribution over the child node states, must sum to one. This ensures that, for example, when all afferent S1 nodes have a flat 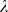 distribution, as in blank regions of the image, the parent C1 node will also show a flat distribution.

Summing up, the steps to learn the weights between simple and complex layers are, for each S1 state 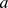 (i.e. each of the four Gabor filter orientations):

1. Find all patches of size 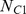 x 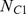 from the 2D matrix given by 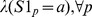, that meet the criterion 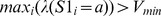, where 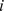 represents the 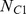
×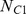 values of the patch and 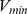 is a threshold value. This ensures that only the patches that contain values above a minimum threshold are taken into account. Given that we are conditioning the patch selection to a specific Gabor filter orientation 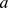, we can consider this step to include aspects of supervised learning.
2. Apply k-means clustering to the selected patches using 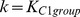, where 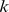 is a fixed parameter representing the number of clusters.

This yields 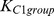 cluster centres of size 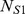
×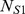 for each of the 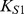 states, making a total of 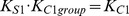 cluster centres or C1 prototypes. Each subset of 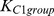 C1 prototypes constitutes a C1 *group*, such that there is one C1 *group* per S1 state. These weights are then converted into the CPTs 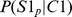 as described above and illustrated in Figure 5. The same learning algorithm is applied between the S2 and C2 layers, but in that case there are 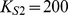 S2 states and C2 *groups*.

To learn the selectivity weights between layers C1 and S2, the *minimum distance* algorithm [13] is employed. First, all potential S2 prototypes 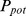, are extracted by sampling from all the locations of the 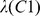 response generated for each of the training images. The number of elements for each prototype is 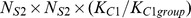, i.e. the S2 RF size times the number of C1 states divided by the states per *group*. As previously stated, to learn the S2 prototypes a single value is used for each *group*, corresponding to the sum over all the C1 states belonging to that *group*. For example, if each C1 node is composed of 40 states divided into 4 *groups* (or invariant features), only the 4 values corresponding to the sum of each *group* of states are used to compute the S2 prototypes. The steps of the *minimum distance* algorithm are the following:

1. The list of selected prototypes, 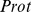, is initialized to contain no prototypes. A parameter called the *minimum distance*, 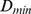, is initialized to a relatively high starting value.
2. Find all patches (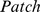), of size 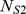
×
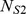
×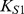 from the 3D matrix given by 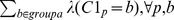, where 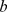 represents the C1 state and 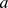 represents the C1 *group* associated with an S1 state. Thus, when learning the S2 prototypes we only consider a single value for each C1 *group*, namely, the sum of its states.
3. Patches are added to the selected prototype list, 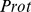, if the Euclidean distance to all previously stored prototypes is above the *minimum distance*, i.e. if 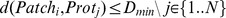 then 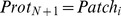, where 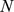 is the number of selected prototypes.
4. Lower 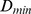 and repeat step 3 until 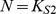. The initial value of 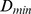 and the decreasing step size in each iteration dictate the dissimilarity between the final selection of prototypes.

The algorithm finds a local optimum in a greedy search sense, aimed at maximizing the Euclidean distance between the extracted prototypes. This algorithm is also used to extract the most common spatial patterns in the Hierarchical Temporal Memory model [13]. In the HMAX model, on the contrary, the prototypes which serve as centres for the Radial Basis Functions are extracted at random from the C1 maps generated by the training images.

As with the prototype weights in the previous layer, these also need to be converted to the CPTs 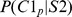 and normalized to conform to probability rules. This is exemplified in Figure 5, which also illustrates a key component of the model, whereby all the weights of C1 states belonging to the same *group* are equal.

The top 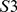 layer employs supervised learning where the weights (prototypes) directly correspond to the output of the C2 layer for each of the object categories. Accordingly, there is a single 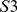 node with 30 states associated with each of the 30 object categories. Given the weight tables for each S3 prototype, it is possible to calculate the final CPTs 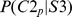 for each C2 node following the conversion method and grouping strategy previously described. This supervised learning stage can be interpreted as mapping the high-level causes of the generative model onto the object category labels, guided by the implicit assumption that human labels are closely related to high level causes.

### Approximations to Simplify the Belief Propagation Operations

This section covers three approximations implemented in the model, which simplify the loopy belief propagation algorithm and allow it to work on a Bayesian network with large dimensions. The first one samples the incoming 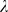 messages to avoid values outside of the system’s numeric range; the second one approximates the output 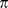 message using the Belief of the node to simplify the generation of messages; and the third one samples incoming 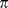 messages to reduce the number of operations (computation time) implemented by each node.

#### 0.0.1 Combining bottom-up messages multiplicatively

Due to the potential large fan-in in the network and the large number of states, calculating the 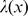 function (Equation (3)) of a node requires multiplying a high number of potentially very low probability values. For example, a node might receive input from 400 (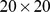 locations) afferent nodes, meaning that it is necessary to obtain the product of 400 probability distributions. The result of this computation is often outside the typical numeric boundaries in simulation environments (for Matlab these boundaries range from 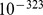 to 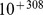). A possible option is to transform the equations to the log domain so that products can be replaced with sums. However, this requires making further approximations for the belief propagation operations that contain weighted sums. For example, [14] describes how the log-sum needs to be approximated with a sum-of-logs. Nonetheless, studying whether these approximations can provide better results than the current ones is an interesting approach for future versions of the model. In the current version of the model we decided to make several approximations to avoid the multiplication problems.

In the first one, the messages (probability distributions) are sum-normalized to one and then re-weighted so that the minimum value of the distribution is never below 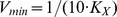. All elements of the message that are below 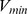 are set to 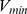. The overall increase in the sum of the elements of the resulting distribution is then compensated by proportionally decreasing the remaining elements (those that were not set to 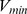). Consequently, the resulting distribution will still be sum-normalized to 1, while having a minimum value equal to 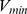. Given that the difference between the values that were below 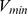 and that 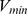 is usually very small, the overall shape of the distribution will remain practically identical to the original one. This adjustment of the message probability distributions ensures all elements are above 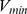, thus allowing multiplicative combination of a greater number of input messages.

The second approximation is defined as follows. Given a node 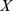 with child nodes 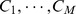, the number of input 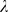 messages is reduced such that 
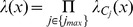
, where 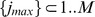, represents the indices of the 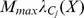 messages with highest variance, and 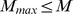. Here, the variance is calculated over the numerical probabilities of the states within the message. The maximum number of input messages, 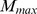, is calculated as a function of the number of states of the messages, 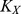, the maximum real value allowed by the system, e.g. 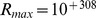, and the minimium value allowed in the probability distributions, 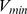, as given by the following equation:
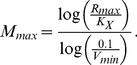
(11)


Thus, the likelihood function of each node is obtained by multiplying only the 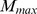 input 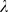 messages with highest variance, where 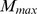 is set to ensure that the result of the computation never reaches the system’s numeric upperbound. The probability distributions with highest variance are chosen as they are likely to carry more information. To implement this approximation Equation (3) is replaced with Equation (12):
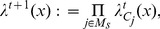
(12)where 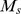 represents the indices of the 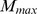 incoming 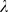 messages with the highest variance.

In the majority of cases 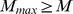, so the resulting equation is identical to that in the original belief propagation formulation. Results measuring the performance of this approximation are included in Text S2. Note that these approximations are aimed at avoiding the numerical range boundaries not at reducing the computation time.

#### 0.0.2 Replacing 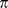 messages with beliefs

As shown in Equation (6), the outward 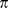 message generated at each node can be obtained as a function of its belief. The only difference is that the message from node 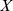 to 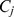, i.e. 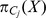, includes all incoming messages to 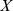, except the one arriving from the destination node, i.e. 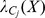.

However, for the purpose of simplification and increased computational performance, and only when the number of incoming messages is high, the outgoing 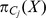 message can be approximated by the belief, 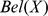. This approximation implies 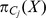 also includes the evidence contained in 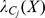. Nonetheless, 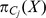 is calculated by combining messages from a total of 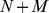 nodes (all parent and children nodes), so the overall effect of one single incoming message on the final output message is proportional to 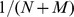. This justifies the approximation in models, such as the one proposed in the example of this paper, where the values of 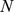 and 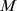 are in the order of hundreds. The same approximation is employed by other similar belief propagation models [13], [15]. To implement this approximation Equation (2) is replaced with Equation (13):
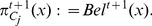
(13)


#### 0.0.3 Reducing the number of operations required to calculate the belief

To reduce the excessive number of operations required to calculate the belief, only the 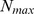 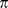 messages, with the highest variance are used in the calculation, where 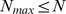. As before, the variance is calculated over the numerical probabilities of the states within the message. Furthermore, for each of the selected 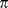 messages, only the 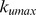 states with the highest values are employed, where 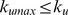. The rationale behind this choice is that the states with the strongest response of the probability distributions with highest variance are likely to carry most of the information content of the parent 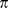 messages. To ensure the belief calculations are still valid it is necessary to select the appropriate columns of the CPTs, i.e. those that correspond to the sampled states of the 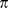 messages. This reduces the number of operations to 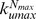 sums and 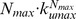 product operations. Although in this section we refer only to the belief calculation, the same method is applied to calculate the 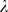 messages, which also integrate information from the parent nodes. Thus, equations (4) and (5) are replaced with equations (14) and (15)
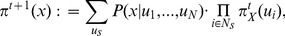
(14)

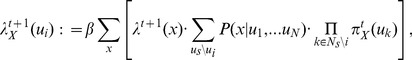
(15)where 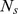 represents the indices of the 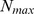 incoming 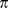 message with highest variance; 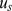 represents the indices of the 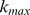 states with highest values out of each of the 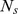 incoming 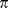 messages.

Results measuring the performance of this approximation are included in Text S2. Note that this approximation is used to generate the Belief (which in turn is used to obtain the output 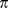 message) and the output 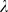 message by sampling and fusing the incoming messages to a node. This recursive process constitutes the backbone of the belief propagation algorithm, so by introducing this approximation the computation time significantly reduced.

### Approximation to Reduce the CPT Size of Nodes with Multiple Parents

This section describes the method employed to approximate the CPTs of nodes with multiple parents and discusses it in the context of other related methods.

Bayesian networks that try to model the visual cortex will inevitably require multiple parent interactions as these arise as a consequence of overlapping receptive fields. The number of elements of the CPT 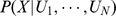 grows exponentially with the number of parents, 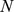, as it includes entries for all possible combinations of the states in node 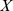 and its parent nodes, e.g. given 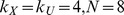, the number of parameters in the CPT is 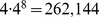, where 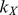 and 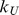 represent the number of states in node 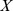 and its parent nodes, respectively.

One common approach to reduce size of the CPTs is based on the concept of independence of causal influences (ICI) [52], [53], which assumes that individual contributions from different causes (parent nodes) are independent and the total influence on the effect (child node) is a combination of the individual contributions. The most standard ICI model is the Noisy-OR [54], [55], which works for boolean or multi-state ordinal variables, those with states that can be naturally ordered (e.g. small, medium, big). However, the Noisy-OR model cannot be applied to categorical variables (e.g. red, green, blue) [55], as is the case with the variables in our network. The states of a node in our model correspond to the different possible features (e.g. four Gabor filters) present at a specific location.

Therefore, in our model, we implement a different method based on the concept of *compatible parental configurations* [33] for expert models, which is closely related to the concept of ICI. This method obtains the final CPT using the weighted sum of simple CPTs. More specifically, it obtains a 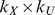 CPT, 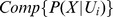, between node 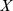 and each of its 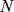 parent nodes, and assumes the rest of the parents, 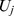, where 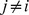, are in *compatible* states (i.e. assumes ICI). More formally, given a node 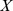 with a set of parents 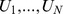, the state 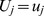 is *compatible* with the state 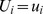, if according to the expert’s mental model the state 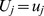 is most likely to coexist with the state 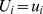. Let 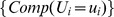 denote the *compatible parental configuration* where 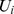 is in the state 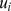 and the rest of the parents are in states compatible with 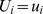.

The method described here proposes combining (using a weighted sum) the CPTS of 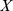, given *compatible parental configurations*, to calculate the CPTs over 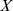, given *incompatible*, or less common, parental configurations. This can be understood as a kind of interpolation mechanism that exploits the known data points. The author [33] makes use of information geometry to demonstrate how these weighted sums capture the experts’ judgemental strategy. A similar ICI model, known as the *average model*, is described in [56].

Although the method was derived for populating CPTs using human experts, theoretically, it can be extended to domains that obtain their information from training data using automatic learning methods. One such domain is hierarchical object recognition, where, due to the great overlap between receptive fields, parent nodes show contextual interdependency and can therefore exploit this technique.

The final CPT 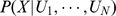 is obtained as a weighted sum of the 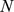 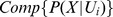 CPTs, where we assume 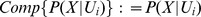. Therefore, the total number of parameters required to be learned grows linearly with the number of parents, more precisely, is equal to 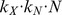. Using the values of the previous example, the number of elements now becomes 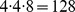, several orders of magnitude smaller than using conventional methods.

After including the CPT approximation for multiple parent nodes, the final equations that replace (14) and (15) are:
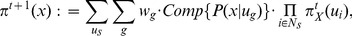
(16)

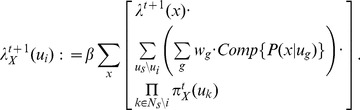
(17)where 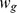 is the weight given to each parent CPT. In our implementation we set 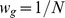, where 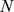 is the number of parent nodes, but in future versions this parameter could be learned during training.

Therefore, the final set of equations implemented in each Bayesian node of the model are: Equations (2) and (6) from the original loopy belief propagation algorithm; and the modified Equations (12), (16), (17) which are approximations to the original algorithm adapted to the proposed large-scale model.

## Results

### Feedforward Processing

This section provides a comparison between the C1 layer response of our model and that of HMAX, demonstrating that our model is capable of approximating the invariance operation. Then the dataset that is used to train and test the model is comprehensively described and the feedforward categorization results are shown for different image distortions (occluded, noisy, translated and scaled) as a function of relevant model parameters. Finally, the model categorization performance is compared to that of two related models, HMAX and HTM, which are tuned using an equivalent procedure to ensure a fair comparison.

**Figure 7:**
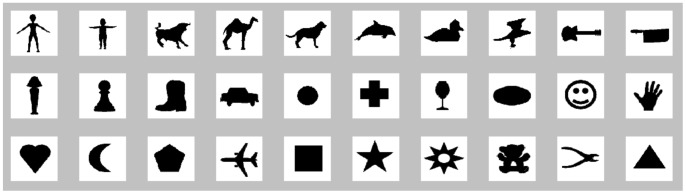
Object dataset. Shows the 30 object silhouette images of 140×140 pixels used to train the model. Transformations of these original images are then used to test the model.

The network was trained using 30 object silhouette images, shown in Figure 7, from which weight matrices were learned. The rationale behind using a custom dataset and not one of the available existing datasets is explained in the Discussion section. The reason for using just one training image per category, sometimes denoted as *one shot learning*, is the fact that the model employs weight sharing. This method simulates the temporal variation of the input that would naturally occur by using dynamic input or by including a mechanism to account for eye saccades, so effectively it is as if the network had been trained with images at all possible locations.

**Figure 8:**
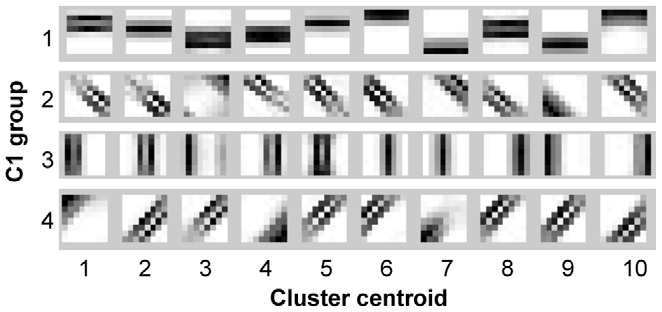
Weight matrices between a C1 node and its afferent S1 nodes. These are learned from the training dataset of 30 object silhouettes following the clustering procedure described, and represent 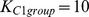 common activation patterns of S1 nodes for each C1 *group*.

The resulting S1-C1 weight matrix, learned from the training dataset of 30 object silhouettes following the k-means clustering procedure described, represent 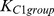 common activation patterns of S1 nodes for each C1 *group*. These are shown in Figure 8 for a value of 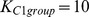. The weights obtained here show very clear and selective patterns where the arrangement of the S1 nodes tends to match the S1 feature orienation, which speaks for a coherence between the local and more global patterns. Note that these weight matrices are the large-scale model analogous of the toy-example weights shown in the left panel of Figure 5, and still need to be transformed into normalized CPTs in order to be used by the model.

**Figure 9:**
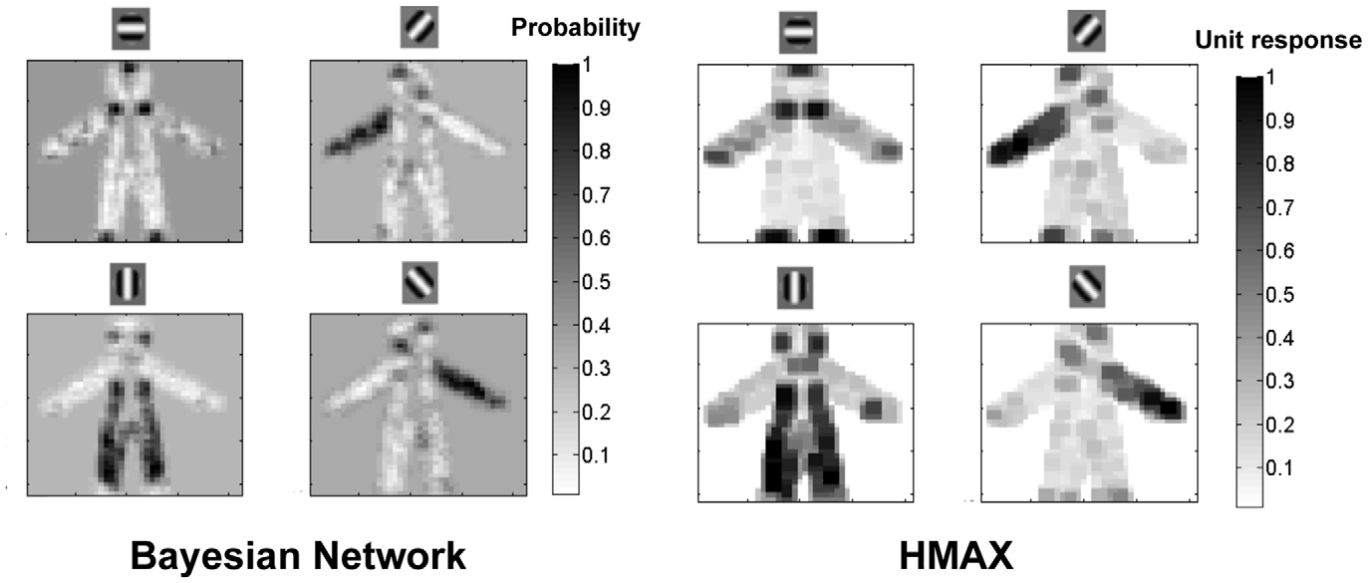
Response of the C1 node in the proposed Bayesian model and the original HMAX model. *left)* Belief response of the C1 nodes, 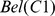. Responses are shown as a 2D map over the locations of the nodes for each *group* of states. The probability for each C1 *group* is calculated as the sum over the probabilities of its constituent states. *right)* Response of the C1 units in the original HMAX model. The response is calculated as the *max* over the S1 afferent units.

Figure 9 compares the feedforward response of the C1 nodes in the Bayesian network, 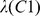, with the response of the C1 units in the original HMAX model. For the Bayesian network, each value represents the sum of the states in each C1 *group* (orientation) at each location, as this is the effective value that will be used to learn the S2 prototypes. The HMAX C1 response is calculated as the *max* over the S1 afferent units, for the same parameter set. The similarity between the HMAX and Bayesian network responses demonstrates that our model is able to successfully approximate the invariance operation. The grey background of the Bayesian network response indicates that features are being coded in a probabilistic manner such that empty regions exhibit equiprobable values for each feature.

In order to test the performance of the model, an image is considered to be correctly categorized when the state with highest probability of the S3 Belief coincides with the input image. Feedforward recognition is performed by assuming initial flat distributions for all the nodes and running belief propagation on the trained network, updating one layer at a time in a bottom-up fashion.

**Figure 10:**
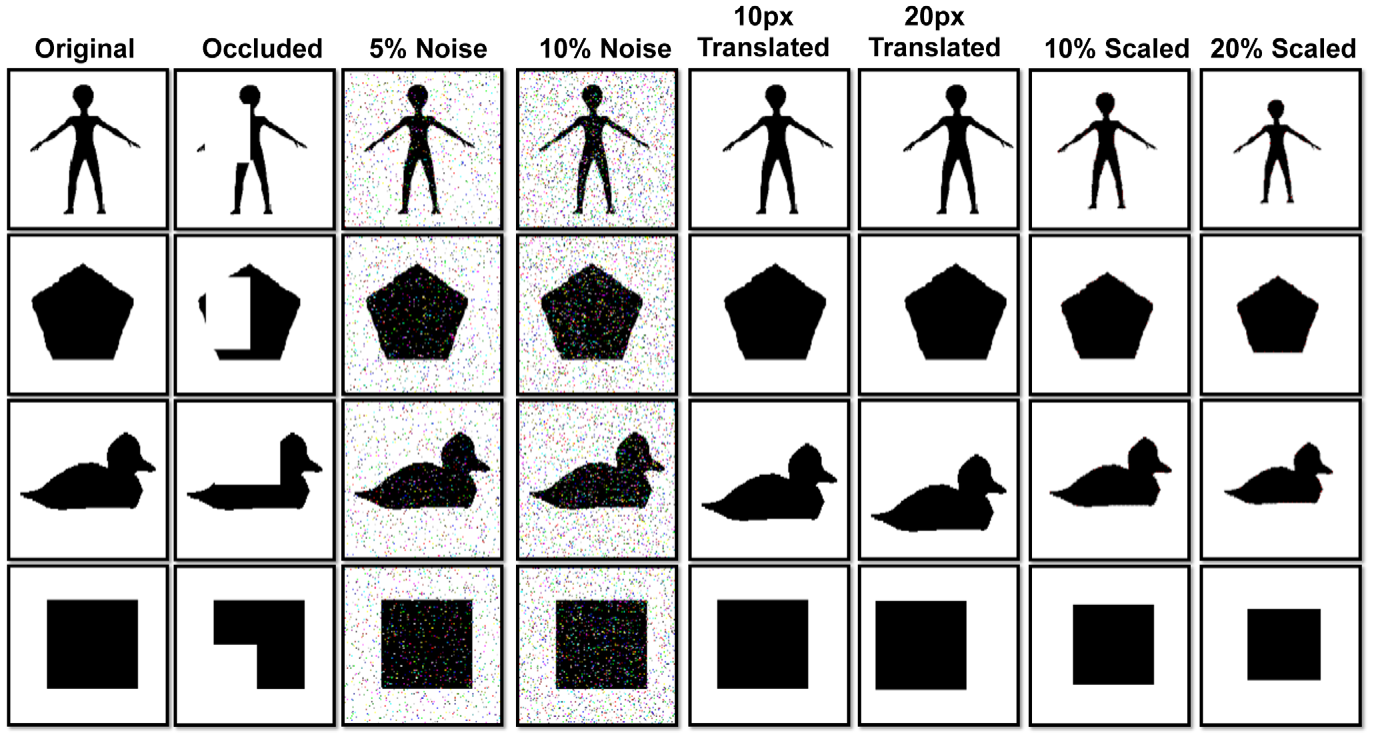
Examples of object transformations. The trained network is tested on different transformations of the training images including occluded, noisy, translated and scaled versions. Examples of these transformations are shown here for four objects.

The model was tested using different distortions of the training images including occluded, noisy, translated and scaled versions, making a total of 1050 testing images (30 categories × 5 variations × 7 distortions). An example of the seven different distortions for four arbitrary categories is shown in Figure 10. Below is a description of how the variations and distortions are generated from the original 30-image training dataset:

- **Occluded**. This distortion involves removing approximately *20%* of the object’s pixels using a rectangular white patch. To generate the five different variations, the rectangle is placed at five different positions in order to occlude different parts of the object.
- **5 and 10 Noise**. For these distortions either *5%* or *10%* of the image pixels change their value to a random number from a uniform distribution. The five different variations are generated by randomly selecting the pixels to change.
- **10 px and 20 px Translated**. These distortions involve moving the the object 10 or 20 pixels away from its original centre position. The five different variations are generated by moving the object up, down, right, left and diagonally (bottom-right) the corresponding number of pixels. Note, in some cases for the 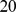 pixel translation, a small part of the object fell outside the image dimensions leading to an additional small occlusion.
- **10% and 20% Scaled**. For these distortions the object is reduced in size either *10%* or *20%* of their original size. To obtain five different variations, the position of the scaled object with respect to the original object was adjusted to either the top-right corner, top-left corner, bottom-right corner, bottom-left corner or centre.

**Figure 11:**
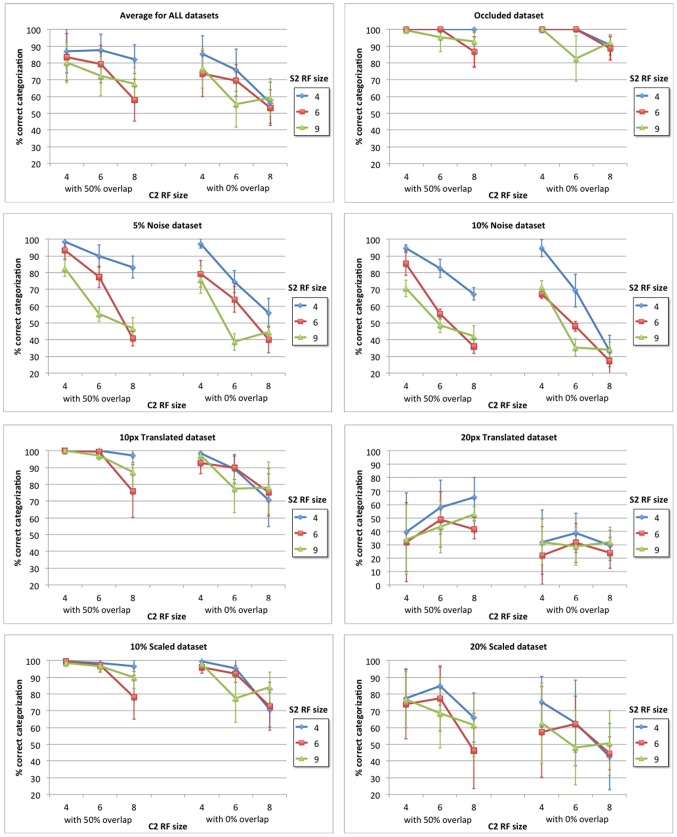
Categorization performance of the model as a function of the S2 receptive field size, 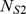 and overlap, and the C2 receptive field size, 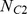. Results show the correct categorization percentage for each distorted dataset, averaged over the five image variations for each distortion. Error bars represent the standard deviation of the correct categorization percentage over the five variations of each distortion. Results are also shown for the average over all distortions. The general trend shows improved performance for smaller S2 and C2 receptive field sizes and for higher C2 receptive field overlap.

**Figure 12:**
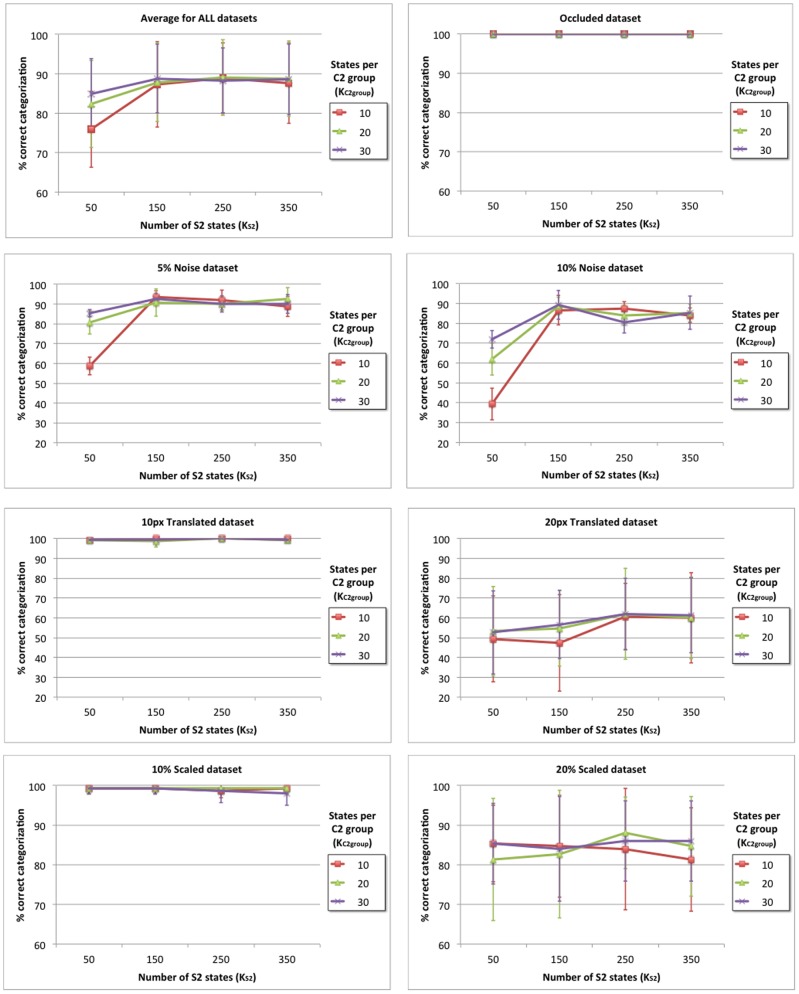
Categorization performance of the model as a function of number of states in the S2 layer, 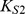 and the number states per *group* in the C2 layer, 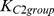. Results show the correct categorization percentage for each distorted dataset, averaged over the five image variations for each distortion. Error bars represent the standard deviation of the correct categorization percentage over the five variations of each distortion. Results are also shown for the average over all distortions. In general the model performance is highly robust to variations of 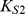 and 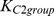, except for the Noisy dataset where a decreased performance is observed when the number of S2 states, 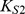, is equal to 50 (lowest value).

**Figure 13:**
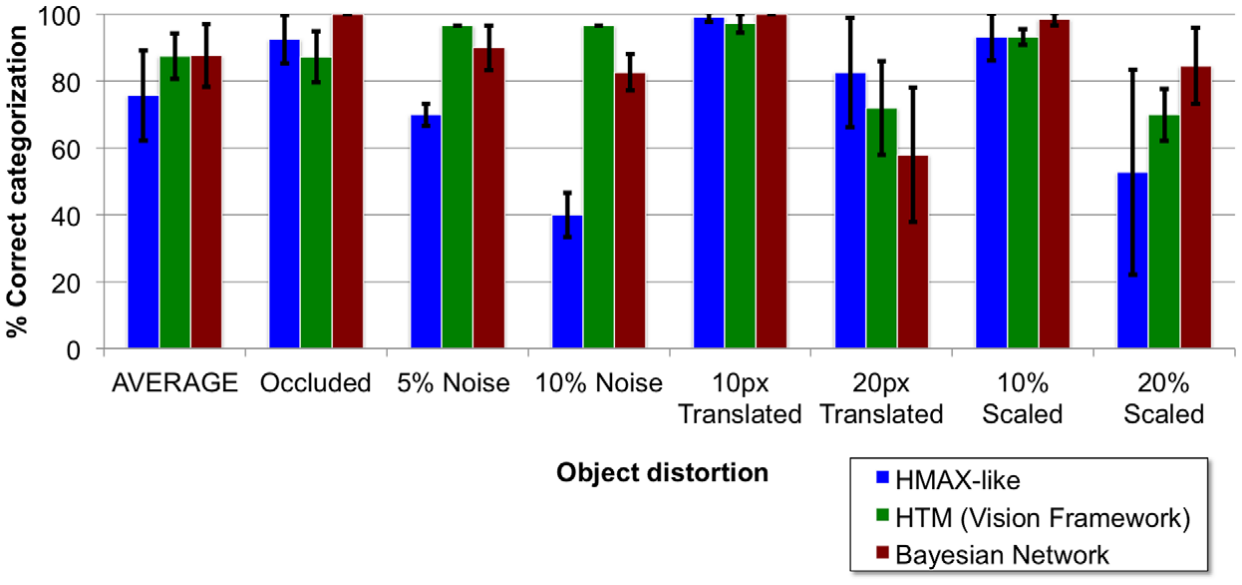
Comparison of categorization performance of the model proposed (Bayesian Network), an HMAX-like model and an HTM network. Results are shown for all the different object distortions averaged over the five image variations within each distortion. Error bars indicate the standard deviation of the correct categorization percentage over the five variations. All models share the same structure parameters including RF sizes, RF overlaps and number of features per layer, except for 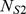, 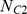 and 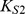, which were tuned independently for each model. Additionally, several model-specific parameters were also optimized to maximize the categorization results. This comparison is only intended to demonstrate that our model can achieve a feedforward categorization performance similar to that of other models. See the text for further details.

We now define a set of simple functions to facilitate the understanding of the different model performance measures used in the result figures. Let 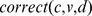 be a boolean function indicating whether the image corresponding to category 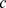 (with range 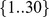), variation 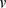 (with range 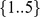) and distortion 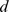 (with range 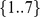) has been correctly categorized (1) or not (0). Let 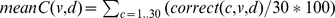 be the correct categorization percentage for variation 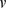 and distortion 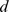. Let 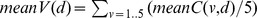 be the mean correct categorization percentage over all variations of distortion 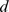. Let 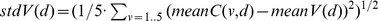 be the standard deviation of the correct categorization percentage over all variations of distortion 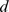. Let 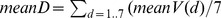 be the mean correct categorization percentage over all variations and distortions. Let 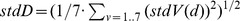 be the average standard deviation, over all distortions, of the correct categorization percentage over all variations. Figures 11, 12 and 13 report the feedforward results using 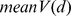 and 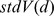 to characterize the performance of the model for each distortion dataset 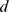; and 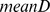 and 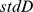 to characterize the performance of the model averaged over all distortion datasets. The specific contents of each figure are described below.

Figure 11 shows the categorization performance of the model for each distorted dataset as a function of the S2 receptive field size (NS2), the S2 receptive field overlap (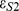) and the C2 receptive field size (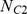). The general trend shows improved performance for smaller S2 and C2 receptive field sizes and for higher C2 receptive field overlap.

Figure 12 shows the categorization performance of the model for each distorted dataset as a function of the number of states in the S2 layer (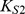) and the number states per *group* in the C2 layers (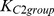). In general the model performance is highly robust to variations of 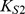 and 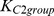, except for the Noisy dataset where a decreased performance is observed when the number of S2 states, 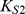, is equal to 50 (lowest value).

Figure 13 compares the categorization performance of 1) an HMAX-like model, 2) a Hierarchical Temporal Memory network and 3) the Bayesian network and belief propagation proposed model. The three models were trained and tested using the same dataset and their structure parameters were tuned over the same parameter space. Additionally, several parameters specific to each model were tuned to maximize the categorization performance. The reason that we cannot compare our results with those of original HMAX and HTM publications, even if we had used the same datasets, is that the structure parameters of the networks would have been different. This is especially significant for published HMAX models, which have several scale bands in each layer, whereas our simplified version has a single scale band per layer.

The HMAX-like model was implemented using Matlab and replicates the model described in [37], the 3-level HMAX implementation, but using the simplified set of parameters shown in Table 1, i.e. with no scale bands. Following the original HMAX implementation, the S2 prototypes are selected at random from the training set, as opposed to employing the *minimum-distance* algorithm implemented in the Bayesian network model. The categorization performance was optimized for the parameter space depicted in Figures 11 and 12, yielding the following optimum values: 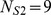, 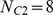 (with 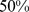 overlap) and 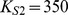. Three other parameters, specific to HMAX, were optimized (the range of parameter values is shown in brackets): the optimum S2 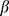 coefficient was 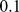 (range  =  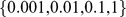); the optimum normalization method for the C2 response was 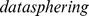 [40] (range  =  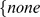, 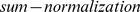, 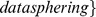); and the optimum SVM kernel was RBF with 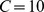 (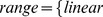, 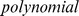, 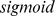, 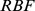 with 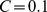, 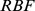 with 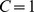, 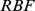 with 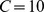, 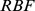 with 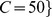).

With respect to the HTM model [13], the network was implemented using the Python-based Numenta Vision Framework and following the structure described in Table 1. Accordingly, layer S1 nodes were set to the 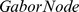 type, layers C1 and C2 nodes to 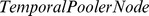 type, layer S2 nodes to 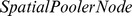 type, and the layer S3 node to 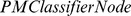. As before, the model performance was optimized for the parameter space illustrated in Figures 11 and 12, obtaining the following parameter values: 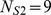, 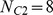 (with 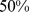 overlap) and 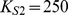. The Vision Framework allows the modification of a large number of HTM-specific parameters, which were set to the standard values used by HTM networks that have been tested on other similar visual datasets, such as the NORB or Fruits dataset. We also note that the model includes auto-tuning function for the top classifier layer, aimed at optimizing the parameters from this layer. Nonetheless, a subset of the HTM-specific parameters, most of them related to the training methods, were tuned to maximize the model performance yielding the following values (range is indicated in brackets): the number of recursions for auto-tuning showed no effect over the performance (range  =  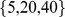); the optimum number of samples per recursion for auto-tuning was 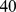 or above (range  =  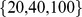); the optimum training method for Layer 2 was 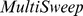, for Layer 3 was 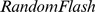, for Layer 4 was 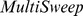, and for Layer 5 was 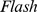 (range = 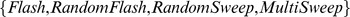).

The comparison between models shown in Figure 13 cannot be used to decide which model deals better with which distortion because some of the structure parameters (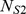, 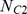 and 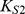), common to all models, have been tuned independently. This means that the models will perform better for some distortions (e.g. higher C2 RF sizes are associated with better performance for the translated distortion) and worse for others, depending on the value of these parameters. However, when the same parameters (e.g. those that give best performance for our Bayesian model) were used for all the models, the average performance of the HTM and HMAX models decreased significantly. The same happened to the average performance of our Bayesian model if the optimum parameters for HTM and HMAX were used instead. Therefore, we decided that the fairest comparison would be provided by showing the categorization performance of each model after maximizing their parameters independently. Overall, the results in Figure 13 suggest that our model can achieve a feedforward categorization performance similar to that of the HTM and HMAX models.

### Feedback Modulation

This section describes results illustrating two feedback effects captured by our model, namely, illusory contour completion and top-down attentional modulation.

**Figure 14:**
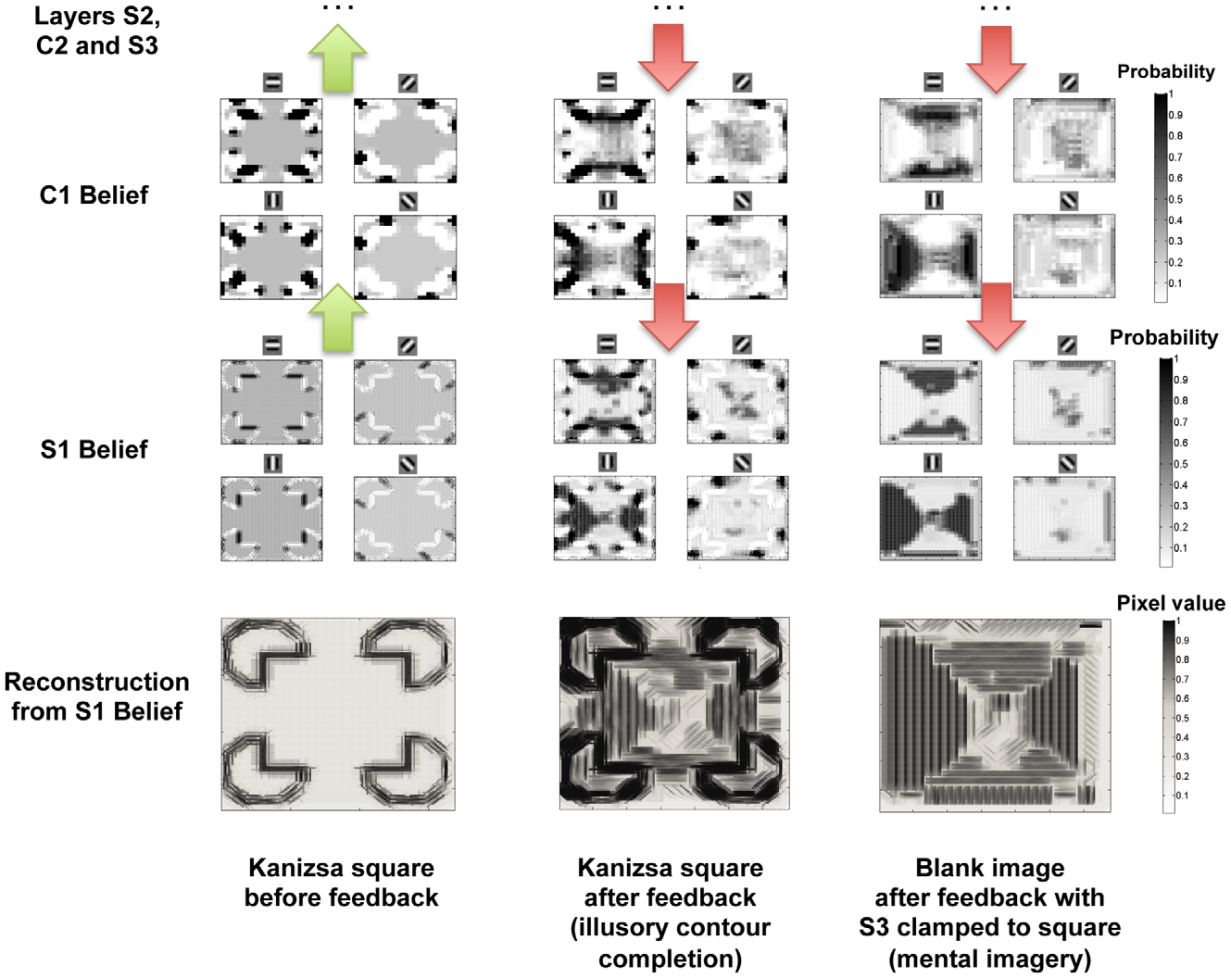
Simulation results reproducing the illusory contour completion and mental imagery phenomena. Image reconstruction and S1 and C1 internal representations for a Kanizsa square input image before *(left)* and after *(middle)* top-down feedback; and to a blank input image, after top-down feedback, with the S3 layer clamped to a square *(right)*. Layers are updated in a sequential up-down fashion in the following order: S1-C1-S2-C2-S3-C2-S2-C1-S1. The representations after top-down feedback are understood as the model responses once the top layer has been updated. The model response to a Kanizsa square input image shows contour completion due to top-down feedback. The model response to a blank input image with square feedback from S3, illustrates the invariant object representation that is being fed back from the top layer in the absence of bottom-up input. This can be understood as reproducing the mental imagery phenomenon. The grey scale indicates the probability of each node being in one of the four states or orientations, signalled by a small oriented Gabor filter at the top of each 2D spatial representation. For the image reconstructions, the grey scale representes the normalized value of the pixel.

To study illusory contour completion in the model, we use a Kanizsa square as input image and allow the internal representation to propagate along the layers, from S1 to S3. Once the input image is categorized as a square in the top layer, the stored square representation is fed back downwards and combined with the bottom layers representations. Figure 14 shows the S1 and C1 internal representations, i.e. the probability of each of the four states (orientations) at every location, before and after top-down feedback. Layers are updated in a sequential up-down fashion in the following order: S1-C1-S2-C2-S3-C2-S2-C1-S1. The representations after top-down feedback are understood as the model responses once the top layer has been updated. Additionally, in order to study the internal representation of a square stored in the S3 layer and to understand how feedback interacts with the existing lower level beliefs, we also show the model response to a blank input image, after top-down feedback, with the S3 node clamped to the ‘square’ state. For each of the three scenarios, an image reconstruction obtained by combining the oriented Gabor filters of the S1 representation is also included. The model parameters for these simulations are the same as those used to obtain the feedforward results and shown in Table 1.

**Figure 15:**
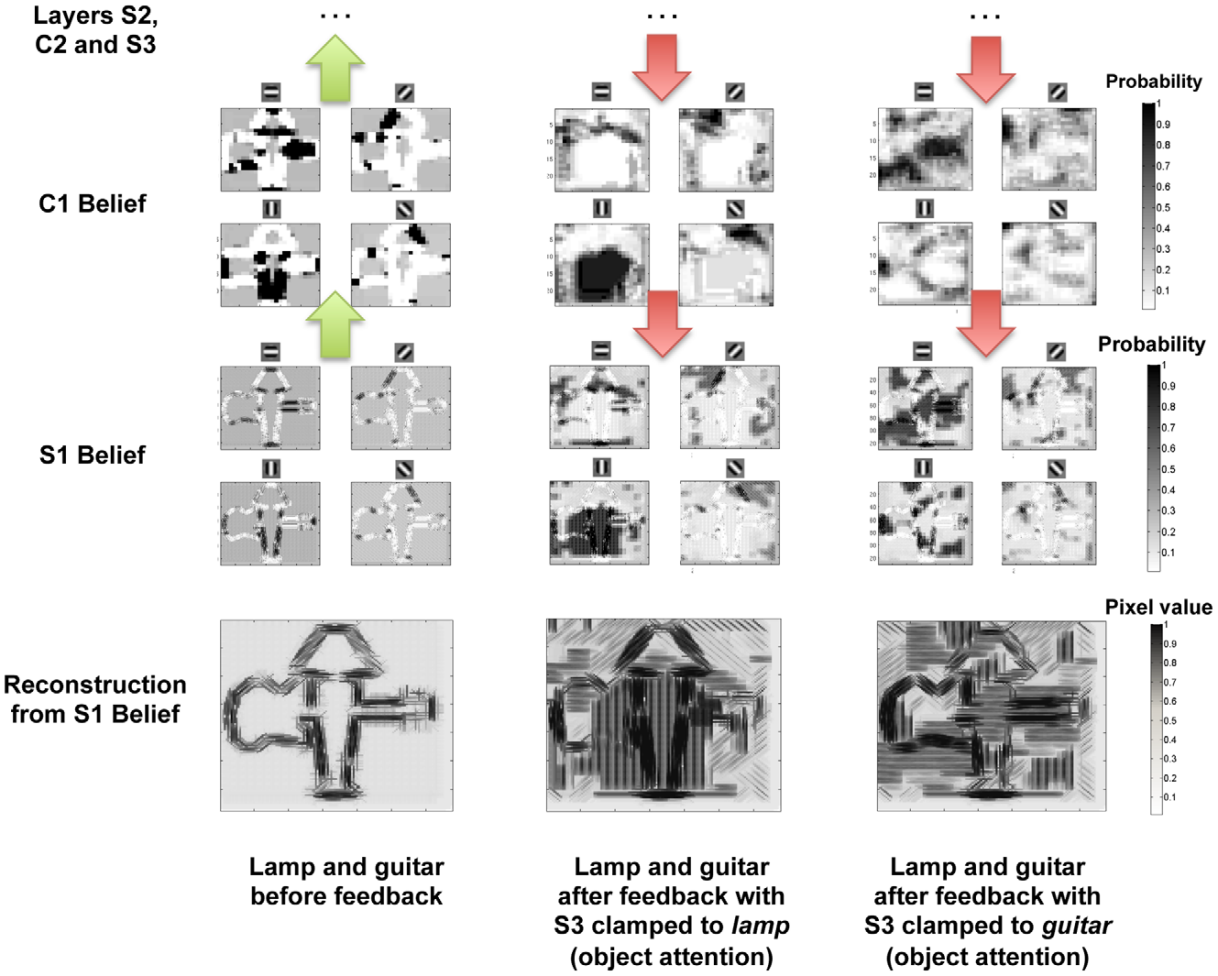
Simulation results reproducing object attention. Image reconstruction and S1 and C1 internal representations for an input image containing a superimposed lamp and guitar, before *(left)* and after top-down feedback with the S3 node clamped to the lamp object *(middle)* and to the guitar object *(right)*. Object attention is simulated by clamping the S3 node to a specific state, the object that will receive top-down attention, and updating the layers in the order S3-C2-S2-C1-S1. The S1 layer will then combine the top-down feedback originated in S3 with the bottom-up sensory data from the input image. The grey scale indicates the probability of each node being in one of the four states or orientations, signalled by a small oriented Gabor filter at the top of each 2D spatial representation. For the image reconstructions, the grey scale representes the normalized value of the pixel.

To simulate object attention in the model, the S3 node is clamped to a specific state, the object that will be attended to, and the layers are updated in a top-down fashion in the order S3-C2-S2-C1-S1. When the abstract object representation reaches the S1 layer it is be combined with the bottom-up sensory information from the input image, and enhances the regions and features of the image that correspond to the attended object. This process is illustrated in Figure 15 for an input image with two superimposed objects, a lamp and a guitar, and two different scenarios simulating top-down object attention on each of the objects.

## Discussion

### Comparison with Previous Models

The recently published CDBN model [32], which extends Deep Belief Networks to a multi-stage Hubel-Wiesel architecture, shares many aspects with our model. They both propose a probabilistic *max-pooling* operation to implement invariance using generative models, learning happens in a bottom-up greedy layerwise fashion, weights are shared amongst nodes in the same layer and results show both feedforward categorization and feedback completion. However, inference in CDBNs is implemented using Gibbs sampling whereas we employ loopy belief propagation together with a number of approximations to simplify the computations. Additionally, CDBNs are based on Restricted Boltzmann Machines, a type of undirected graphical model with binary states, whereas our model is based on Bayesian networks. As a consequence each CDBN node represents a binary variable encoding a specific feature and location while our Bayesian network nodes represent multiple-state variables encoding the probability distribution over features for a given location. The invariance operation in CDBNs is implemented by dividing the selectivity simple nodes into disjoint blocks that feed into each *max-pooling* complex node and constraining to one the maximum number of active simple nodes. In our model each complex node learns the most common activation patterns of afferent nodes for each simple feature and *groups* them during the generation of the output message to the layer above, thus approximating the *max-pooling* operation. Our method is not limited to disjoint blocks of afferent nodes, such that a simple node may have multiple parents (overlapping receptive fields). Learning in CDBNs happens through the contrastive divergence approximation as opposed to the non-probabilistic discriminative methods (minimum distance and k-means algorithms) employed by our model, which require the subsequent conversion to normalized conditional probability tables.

HTM networks [13] also constitute an example of a probabilistic model with a multi-stage Hubel-Wiesel architecture. Furtheremore, the way in which complex features are constructed is similar to our model in the sense that they represent *groups* of simple features. However, there are significant differences with our model, starting with the fact that HTMs combine simple and complex features into a single node and so they cannot be understood as a conventional Bayesian network. Consequently, HTMs require a significantly modified belief propagation algorithm adapted to this special type of node. Our proposed model retains the conventional definition of a Bayesian node which allows us to model simple and complex units of HMAX-like models using individual Bayesian nodes in separate layers. Additionally, the solution proposed by [13] to deal with multiple parents, the Noisy-OR gate, is not valid for the type of variables in most HTMs (categorical variables). Finally, the authors suggest using loopy belief propagation but do not show any examples or results, thus omitting the critical problem of implementing networks with loops.

The model proposed by Ullman [57] implements exact inference using belief propagation. However, it employs over-simplified tree-structured networks with no loops and is qualitatively different from the proposed model in that the nodes correspond to features and their states to locations. Furthermore, the model requires one independent network for each object category. Similarly, the model described in [58] also implements exact inference on a singly-connected Bayesian network but models exclusively high-level attention, where the lower half of the network that performs recognition is non-Bayesian and strictly feedforward.

Other related models remain purely theoretical [47] or employ different methods to perform approximate inference, such as variational approximations [6], [19]. The variational approximation method tries to minimize the free-energy of the system, by minimizing the difference between the approximate or recognition distribution and the true posterior distribution. In the model proposed by Friston [6], this is achieved by implementing a message-passing algorithm that solves the equations of a hierarchical dynamic network. This local and recursive message-passing scheme is reminiscent of belief propagation but individual messages do not correspond to probability distributions and are more difficult to interpret.

### Similarities with the Visual Cortex

As has been extensively argued in the literature, the parallel, distributed and hierarchical architecture of the cortex has significant similarities with the structure of Bayesian networks [5], [13], [54]. Furthermore, the homogeneous internal structure of cortical columns (the canonical microcircuit) is comparable to the homogeneous internal operations (belief propagation) of each Bayesian node. This has lead to the proposal of possible cortical mappings and biologically plausible implementations of belief propagation [6], [13], [15], [16], [59]. Additionally, the Bayesian network proposed here has an architecture similar to that of the HMAX model, which has been shown to capture widely accepted principles of object recognition in the ventral path of the visual system [31], [41].

Although the approximations of the model were mainly intended to make the model work on a real dataset and with HMAX-like parameters, some of them can be justified from the neurobiological perspective. For example, the use of loopy belief propagation is consistent with the known recurrent connectivity of neuronal networks in the visual cortex; and the sampling of incoming messages is consistent with the subthreshold activity of many neurons and the consequent high degree of sparseness observed in spiking patterns [60].

There is always a trade-off between the biological realism of a model and its large-scale functionality. In this study we focus on the latter, in contrast to a number of papers have already provided detailed biological implementations [15], [16], [59] but limited to small-scale toy examples.

### Feedforward Processing

First of all, it is important to highlight the importance of the HMAX multi-stage Hubel-Wiesel architecture that has been reproduced by the Bayesian network proposed. Recent feedforward ConvNet models, which employ this type of architecture, have achieved the best published results on well-known benchmarks for object classification (NORB, CIFAR10, Caltech101) [61]–[63] and handwritten digit recognition (MNIST) [61]. Although here we have focused on the HMAX model, our methodology can be potentially applied to build Bayesian networks with belief propagation capable of reproducing the structure and functionality of other HMAX-like models, such as ConvNets.

For the feedforward results in this study we wanted to use a dataset with the following characteristics: 1) it allowed the model to be tested on a specific and differentiated set of distortions (occlusion, noise, translation and rescaling); 2) it had a moderate number of images, allowing us to tune the model parameters despite being a computationally demanding model; 3) the size of the images was large enough to ensure that the task was not trivial and that the model could be potentially extended to larger natural images; and 4) the images were simple enough that they allowed us to clearly test feedback effects such as illusory contour completion.

Initially we considered using one of the existing datasets, but none of them satisfied all of these criteria. The available datasets either didn’t contain distorted versions of the images but had many examples of each category (*Caltech* [64], *USPS* [65] and *MNIST* [66]); the distortions were beyond the scope of our model (rotations in *SDIGIT* [67] and 3D transformations in *NORB* [68]); or the image size was very small (16×16 px for *SDIGIT* and *USPS*, 28×28 px for *MNIST* and 32×32 px for *Pictures* [69]). For these reasons we decided to generate our own dataset, matching the described criteria, with a strong focus on clearly illustrating feedback effects. Other authors have previously raised concerns with many of these datasets, arguing they didn’t appropriately capture their problem of interest, and have also chosen to generate an independent dataset [63].

The categorization results in Figure 11 show some clear patterns: performance improves for smaller S2 and C2 receptive fields and higher S2 receptive field overlap, at least within the limited range of the values tested. Smaller receptive fields provide higher selectivity, but presumably, lower values would at some point decrease the performance as the invariance capability of the model deteriorates. An exception occurs for the 20 px translated dataset where the performance increases proportionally with the C2 receptive field size. This suggests small C2 pooling regions are able to cope with all other distortions and therefore benefit from the improved selectivity, but fail to account for the 20 px translation. The overall robustness of the model as a function of the S2 and C2 receptive field sizes is relatively high (maximum average difference of approximately 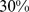), but can vary significantly for different distortions (e.g. high robustness for the Scaled dataset but relatively low for the Noise dataset).

Similarly, the categorization results in Figure 12 suggest that, within the parameter range tested, the model is highly robust to the number of states in the S2 layer and the number of states per *group* in the C2 layer. The performance only slightly decreases for the case when the number of S2 states is 50 (minimum value), but otherwise stays around an average value of approximately 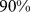. Finally, Figures 11 and 12 also indicate that the robustness of the model to quantitative increases of each distortion (e.g. noise level between 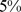 and 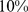) is relatively good for the noise dataset but not for the scaled and translated one.

The standard deviation over the five variations of each distortion (see Results section for details), shown as error bars in Figures 11, 12 and 13, is relatively low for all distortions except for the *Translated 20 px* and *Scaled 20%*. This is because the five variations for each image involved moving the translated or scaled image to a different position and the model seems to be sensitive to the direction of displacement, e.g. scaled objects that are moved to the top-right corner are more difficult to categorize than those at the bottom-right corner. Consequently, the statistical significance of the results obtained for these two distortions is low. Similar high standard deviations are observed in both the HTM and HMAX categorization results (Figure 13).

The comparison of results in Figure 13 demonstrates that the proposed Bayesian network can achieve competitive feedforward categorization results, comparable with those of similar models, such as HMAX and HTM. The structure parameters, including pooling and step sizes and number of features for each layer, were identical for the three models, except for three parameters: 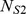, 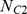 and 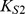. These parameters were optimized independently for each model, over the same parameter space used for our model (shown in Figures 11 and 12). Additionally, a significant number of model-specific parameters were tuned to maximize the categorization performance of both HMAX and HTM. We also note that it is very likely that further parameter tuning could have improved their categorization results, but the same applies for our model. Similarly, using extended versions of HTM [67] or HMAX [40] could also yield better performance, but this study we decided to focus on their original published versions. Ultimately, we would like to strongly emphasize that this comparison is only intended to illustrate that the feedforward capabilities of our model are similar to those of related models, but not to demonstrate superiority in terms of object recognition for artificial vision systems. The focus of this paper is to show a Bayesian-based feedback extension of the HMAX model consistent with evidence from the visual cortex.

### Feedback Processing

The proposed network naturally extends hierarchical feedforward models, such as HMAX, to include dynamic and recursive feedback, which has been pinpointed as the main limitation of these models by their authors [37]. This is made possible through the approximation to the selectivity and invariance operations using belief propagation which also serves to feed back and integrate top-down information. Furthermore, the methods proposed facilitate the implementation of belief propagation in large-scale Bayesian networks where nodes have multiple parents.

First of all, to reduce the size of the connectivity matrices we particularize the weighted sum method proposed by [33] to the visual domain. This method has a strong advantage over the Noisy-OR gate [54], a widely used method that is limited to ordinal variables. The proposed method can be applied to categoric variables with no given order, such as the visual features encoded at each location.

Additionally, to reduce the exponential growth of the number of operations we propose sampling the probability distributions of the incoming messages. We demonstrate empirically that this method provides a good fit to the exact distributions given a moderate number of samples (see Text S2). Nonetheless, it would be interesting to quantify the loss of information due to the sampling methods and investigate how this affects both feedforward and feedback processing. This loss of information may affect the temporal evolution of the response, which, in many cases converges during the first time steps, i.e. the Belief either does not vary significantly over time or oscillates between two fixed points. This may prevent the model from exhibiting further interactions between bottom-up and top-down information.

One key aspect that could help to minimize the loss of information due to sampling in the model is the sparseness of the node activations. The average sparseness at each layer, measured as the percentage of elements in each node with a probability above 1/K (where K  =  number of states), is 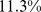 for S1, 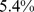 for C1, 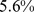 for S2, 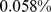 for C2 and 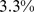 for S3. We hypothesize sparseness arises in the model naturally as a consequence of the multiplicative combination of the large number of incoming 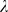 messages at each node. However, further enforcing sparse representations, which have been identified as an essential element for similar biologically-inspired models [13], [19], [30], [32], may improve the accurary of the model approximations.

The results in Figure 14 illustrate an emergent property of the model by which bottom-up evidence from the input image is recursively combined with top-down information leading to image reconstruction or illusory contour completion. There are several important aspects to highlight here. First of all, the phenomenon occurs without any external artifact related to the feedback generation. In other words, the square feedback arises naturally from the S3 layer after the Kanizsa figure is recognized as a square. This means the information that is fed back corresponds to that of the abstract invariant representation of a square stored in the upper layers. This high-level representation can be clearly observed by clamping the S3 layer to the ‘square’ state and leaving blank the input and the rest of the layers (third scenario in Figure 14). By comparing this to the case where a Kanizsa square is used as input image, the interactions between top-down feedback and bottom-up input become apparent: the high-level square representation is refined by the lower-level local information.

These simulation results are consistent with the qualitative response pattern observed across the ventral system, where the Kanizsa figure is represented as a complete figure in the higher levels [70], [71] and, as time progresses, an activation, weaker than that of real contours, can be observed in lower levels [72]–[76]. The model is also consistent with the mechanisms proposed to be responsible for contour completion [77], namely, figural feedback and lateral interactions. Although there are no explicit lateral connections in the model, these are implemented implicitly by the bottom-up messages and top-down messages, both of which take into account evidence from nodes adjacent to the target. Finally, the results in Figure 14 (right) are also in agreement with evidence suggesting that the same visual pathways are shared for visual perception and mental imagery, resulting in similar cortical activations [78], and that mental imagery can lead to retinotopic activations in lower level visual regions [79].

The example shown in Figure 15 serves to illustrate the capacity of the model to simulate top-down feedback modulation of the lower layers, by modifying the S3 node to reflect the appropriate bias towards certain objects or locations. These biases can be understood as attentional, priming or expectation effects, which can arise from areas outside the ventral pathway such as the dorsal pathway [80], prefrontal cortex, fusiform gyrus, posterior parietal cortex or the amygdala [81], [82]. Simulation results are consistent with studies showing the modulation of lower-level regions due to feedback from object-related regions, such as the inferotemporal cortex [2] and the lateral occipital complex [3]. Importantly, these effects are accommodated as part of the Bayesian network mathematical framework, without the need to include any external artifacts. For larger input images containing several objects, the model could potentially implement spatial attention in a similar fashion by defining a prior distribution that favours certain locations.

### Future Work

The model could benefit from a more detailed analysis of how different learning schemes, including online adaptation, as well as different message-passing scheduling methods, affect the categorization and feedback processing. Unsupervised learning methods could also be used for the lower layer, replacing the current HMAX-like hard-wired Gabor filters, which could provide more precision in the feedback reconstruction. Additionally, an interesting extension could come from adding a backpropagation fine-tuning stage, similar to that of deep belief networks [17], to improve the categorization performance.

Another important aspect is the scalability of the model, which is limited by the high computation time required to train the network making it infeasible to run on large datasets. However, the model is still in its infancy and as new learning methods, belief propagation optimizations and more computational power become available this limitation will disappear and the advantages of probabilistic generative models over conventional feedforward models will only increase. In this line, it is important to highlight that the proposed model is well-suited for real-time, parallel and distributed hardware implementations [24].

Finally, an interesting future line of research will be to adapt the proposed framework to other scenarios with similar hierarchical perceptual properties, such as cortical auditory processing.

## Supporting Information

Text S1 **Layer by layer description of the HMAX model**. Describes the operations, including the equations, performed at each of the five layers of the original HMAX model.
(PDF)

Text S2
**Empirical results for the proposed approximations**. Provides results supporting the validity of the approximations used to calculate the 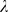 and 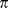 messages. These approximations involve sampling the incoming messages to a node. The results are obtained by calculating the Kullback-Leibler divergence between the true and approximate distributions for a generic node as a function of several parameters.(PDF)
