# Supplementary material for: Top-Down Feedback in an HMAX-Like Cortical Model of Object Perception Based on Hierarchical Bayesian Networks and Belief Propagation
Source: PLoS One. 2012 Nov 5;7(11):e48216. doi: 10.1371/journal.pone.0048216 (PMC3489785; doi:10.1371/journal.pone.0048216)
Supplement: Text S1 — Layer by layer description of the HMAX model. Describes the operations, including the equations, performed at each of the five layers of the original HMAX model. (PDF) [file pone.0048216.s001.pdf]

Supplementary Text S1: Layer by layer description of the HMAX model

Salvador Dura-Bernal[1,*], Thomas Wennekers[2], Susan L. Denham[2]
1 Department of Physiology and Pharmacology, State University of New York Downstate Medical Center, Brooklyn, NY, USA
2 Cognition Institute, University of Plymouth, Plymouth, Devon, UK
∗ E-mail: salvadordura@gmail.com

S1 layer - Units in this layer implement Gabor filters, which have been extensively used to model simple cell receptive fields (RF), and have been shown to fit well the physiological data from striate cortex [1]. There are 64 types of units or filters, one for each of the $K_{S1}(=4)$ orientations $(0°, 45°, 90°, 135°)$ x $\Delta N_{S1}(=16)$ sizes or peak spatial frequencies (ranging from 7x7 pixels to 37 x 37 pixels, in steps of 2 pixels). The four different orientations, although an oversimplification, have been shown to be sufficient to provide rotation and size invariance at the higher levels. Phases are approximated by centring the Gabor filters at all locations. The input image, a gray-valued image is filtered at every location by each of the 64 Gabor filters described by the following equation:

$$G_{x,y} = \exp\left(-\frac{(x\cos\theta + y\sin\theta)^2 + \gamma^2(-x\sin\theta + y\cos\theta)^2}{2\sigma^2}\right) \times \cos\left(2\pi\frac{1}{\lambda}(x\cos\theta + y\sin\theta) + \phi\right) \quad (1)$$

The parameters in the equation, that is, the orientation θ, the aspect ratio γ, the effective width σ, the phase ϕ and the wavelength λ, determine the spatial receptive field of the S1 units. These parameters were adjusted so that the tuning profiles of S1 units match those of V1 parafoveal simple cells in monkeys [2].

C1 layer - Units in this layer correspond to cortical complex cells showing a bigger RF size and a certain degree of position and size invariance. Each C1 unit receives input from a $\Delta N_{C1} \times \Delta N_{C1}$ square array of retinotopically organized S1 units with the same orientation, thus preserving feature specificity. C1 units are arranged in 8 scale bands, where units at each scale band pool from two S1 RF sizes, e.g. C1 scale band 1 pools from S1 units with RF sizes 7 and 9. The pooling grid size, ΔN_{C1}, ranges from 8 pixels to 22 pixels, in steps of 2 pixels, according to the C1 scale band. The *max-pooling* operation is implemented, i.e. the activity of each C1 unit is determined by the strongest of its ($\Delta N_{C1} \times \Delta N_{C1}$ positions $\times$ 2 RF sizes) afferent S1 units. This is shown in the following equation:

$$C1_{b_{C1},x_{C1},y_{C1},k} = \max_{b_i,x_i,y_i} \left(S1_{\{b_i,x_i,y_i\},k}\right) \quad (2)$$

where k represents the feature (in this case the filter orientation),

b_{C1}, x_{C1}, y_{C1} represents the band and location of the C1 unit,

$\{b_i, x_i, y_i\}$ represents the band and location of the afferent S1 units, and are given, as a function of the C1 unit's band and location and the network parameters, by the following expressions:

$$b_i \in \{2 \cdot b_{C1} - 1\ ,\ 2b_{C1}\} \quad (3)$$

$$x_i \in \{1 + (x_{C1} - 1) \cdot \epsilon_{C1}(b_{C1}), \cdots, 1 + (x_{C1} - 1) \cdot \epsilon_{C1}(b_{C1}) + \Delta N_{C1}(b_{C1})\} \quad (4)$$

$$y_i \in \{1 + (y_{C1} - 1) \cdot \epsilon_{C1}(b_{C1}), \cdots, 1 + (y_{C1} - 1) \cdot \epsilon_{C1}(b_{C1}) + \Delta N_{C1}(b_{C1})\} \quad (5)$$

This means each C1 unit represents a Gabor-like feature of the same orientation as the S1 units that feed into it, but with a certain position and size invariance. Additionally, C1 units implement contrast invariance, mimicking complex cells in striate cortex, by taking the absolute value of their S1 inputs.

Therefore, at each C1 location there are 32 C1 units, one for each of the $K_{C1}(=4)$ orientations $\times$ 8 scale bands. Note that, unlike S1 units, C1 units are not computed at every possible location but are sampled every ϵ_{C1} pixels or S1 units, where ϵ_{C1} ranges from 3 pixels to 15 pixels, in steps of 2 pixels, according to the C1 scale band.

Physiological data on simple and complex RF size, spatial frequency and orientation bandwidth are in good agreement with the model S1 and C1 tuning properties, as well as with the hypothesis of complex cells performing a *max-pooling* operation over simple cell afferents [2].

S2 layer - The response of each S2 unit depends in a Gaussian-like way on the Euclidean distance between the input and previously learned prototypes. More specifically, it implements a Radial Basis Function (RBF) network, where the prototypes are the RBF centres. During the training phase, K_{S2} prototypes are learned from the C1 layer, each one composed of $\Delta N_{S2} \times \Delta N_{S2} \times K_{C1}(=4)$ elements, where $K_{S2} = 2000$ and $\Delta N_{S2} = 3$, which yields 2000 prototypes with $3 \times 3 \times 4 = 36$ elements.

During the recognition phase, the response of an S2 unit at a particular location and coding a specific learned prototype or RBF centre is calculated as the distance between the input patch of $\Delta N_{S2} \times \Delta N_{S2}$ C1 units, and the kth stored prototype P_k, such that,

$$S2_{b_{S2},x_{S2},y_{S2},k} = \exp\left(-\beta \cdot \left|\left|C1_{\{b_i,x_i,y_i\}} - P_k\right|\right|^2\right) \tag{6}$$

where β is the square of the inverse width of the RBF and therefore defines the sharpness of the tuning curve,

b_{S2}, x_{S2}, y_{S2} represents the band and location of the S2 unit,

$\{b_i, x_i, y_i\}$ represents the band and location of the afferent C1 units, and is given, as a function of the S2 unit's band and location and the network parameters, by the following expressions:

$$b_i = B_{S2}$$
$$x_i \in \{x_{S2}, \cdots, x_{S2} + \Delta N_{S2}\} \tag{7}$$
$$x_i \in \{y_{S2}, \cdots, y_{S2} + \Delta N_{S2}\} \tag{8}$$

C2 layer - In the C2 layer, units perform the *max-pooling* operation pooling over a $\Delta N_{C2} \times \Delta N_{C2}$ square lattice of S2 units tuned to the same preferred stimulus, i.e. the same learned prototype. C2 units are therefore selective to the same stimulus as their S2 input units but present an increased position invariance. At each location, C2 units will code each of the $K_{C2} = K_{S2}$ learned prototypes, which can now be considered position invariant prototypes. In the HMAX version we are describing [3], ΔN_{C2} is set such that a single C2 unit for each prototypes receives input from S2 units at all locations and scale bands tuned to the same prototype. Other versions [4] use smaller values of ΔN_{C2} leading to multiple C2 units. It has been shown that the S2-C2 hierarchy produces both selectivity and invariance parameters that match observed responses in V4 [5].

S3 layer - This constitutes the top layer of the model and implements a support vector machine (SVM) that uses the K_{C2} output features from the C2 layer to learn, in a supervised manner, the objects or input images. Using a training set of images, the weights of the support vector machine are adjusted in order to classify the output C2 features generated by the input images into the different learned object categories.

References

[1] Jones JP, Palmer LA (1987) An evaluation of the two-dimensional gabor filter model of simple receptive fields in cat striate cortex. Journal of Neurophysiology 58: 1233-1258.

[2] Serre T, Riesenhuber M (2004) Realistic modeling of simple and complex cell tuning in the hmax model, and implications for invariant object recognition in cortex. Massachusetts Institute of Technology, Cambridge, MA CBCL Paper 239/AI Memo 2004-017.

[3] Serre T, Wolf L, Bileschi S, Riesenhuber M, Poggio T (2007) Robust object recognition with cortex-like mechanisms. IEEE Transactions on Pattern Analysis and Machine Intelligence 29: 411-426.

[4] Serre T, Oliva A, Poggio T (2007) A feedforward architecture accounts for rapid categorization. Proceedings of the National Academy of Sciences 104: 6424-6429.

[5] Cadieu C, Kouh M, Pasupathy A, Connor CE, Riesenhuber M, et al. (2007) A model of V4 shape selectivity and invariance. Journal of Neurophysiology 98: 1733-1750.
