# Supplementary material for: Top-Down Feedback in an HMAX-Like Cortical Model of Object Perception Based on Hierarchical Bayesian Networks and Belief Propagation
Source: PLoS One. 2012 Nov 5;7(11):e48216. doi: 10.1371/journal.pone.0048216 (PMC3489785; doi:10.1371/journal.pone.0048216)
Supplement: Text S2 — Empirical results for the proposed approximations. Provides results supporting the validity of the approximations used to calculate the and messages. These approximations involve sampling the incoming messages to a node. The results are obtained by calculating the Kullback-Leibler divergence between the true and approximate distributions for a generic node as a function of several parameters. (PDF) [file pone.0048216.s002.pdf]

Supplementary Text S2: Empirical results for the proposed approximations

Salvador Dura-Bernal[1,*], Thomas Wennekers[2], Susan L. Denham[2]
1 Department of Physiology and Pharmacology, State University of New York Downstate Medical Center, Brooklyn, NY, USA
2 Cognition Institute, University of Plymouth, Plymouth, Devon, UK
∗ E-mail: salvadordura@gmail.com

The original belief propagation equations are shown below:

$$Bel^{t+1}(x) = \alpha \cdot \lambda^{t+1}(x) \cdot \pi^{t+1}(x) \tag{1}$$

$$\lambda^{t+1}(x) = \prod_{j=1..M} \lambda^{t}_{C_j}(x) \tag{2}$$

$$\pi^{t+1}(x) = \sum_{u_1,...,u_N} P(x|u_1,...,u_N) \cdot \prod_{i=1..N} \pi^{t}_{X}(u_i) \tag{3}$$

$$\lambda^{t+1}_{X}(u_i) = \beta \sum_{x} \left[\lambda^{t+1}(x) \cdot \sum_{u_1,...,u_N \backslash u_i} P(x|u_1,...u_N) \cdot \prod_{k=1..N\backslash i} \pi^{t}_{X}(u_k) \right] \tag{4}$$

$$\pi^{t+1}_{C_j}(x) = \alpha \prod_{k \in M \backslash j} \lambda^{t}_{C_k}(x) \cdot \pi(x) = \alpha \cdot \frac{Bel^{t+1}(x)}{\lambda_{C^t_j}(x)}. \tag{5}$$

The approximation related to the λ messages is shown below (see main text for details):

$$\lambda^{t+1}(x) := \prod_{j \in M_s} \lambda^{t}_{C_j}(x) \tag{6}$$

The approximation related to the π messages is shown below (see main text for details):

$$\pi^{t+1}(x) := \sum_{u_s} P(x|u_1,...,u_N) \cdot \prod_{i \in N_s} \pi^{t}_{X}(u_i) \tag{7}$$

$$\lambda^{t+1}_{X}(u_i) := \beta \sum_{x} \left[\lambda^{t+1}(x) \cdot \sum_{u_s \backslash u_i} P(x|u_1,...u_N) \cdot \prod_{k \in N_s \backslash i} \pi^{t}_{X}(u_k) \right] \tag{8}$$

To check how well the proposed sampling procedure (Equation (6)) managed to approximate the exact likelihood functions (Equation (2)) the method was tested statistically by simulating a set of generic child nodes sending messages to its parent node. Using randomly generated λ messages, the difference between the exact likelihood and the approximated likelihood distribution obtained after sampling was measured. The difference was measured using the Kullback-Leibler (K-L) divergence which calculates the cross-correlation between an approximate distribution and the true distribution. Figure 1 shows the K-L divergence between the true and approximate likelihood distributions, averaged over 500 trials, as a function of M_{max}, the total number of child nodes M, and the number of states, k. For comparison, the K-L divergence between the exact likelihood and a randomly generated likelihood distribution is

also plotted. The λ messages were generated by sum-normalizing a set of random values extracted from the following distribution: $|\mathcal{N}(10, |\mathcal{N}(0, 5)|)|$. This simulates the distribution pattern observed in the λ messages of the model, many of which will typically present near-flat distributions as they originate from blank regions of the image.

The results show that the goodness of fit between the approximation and the exact solution increases as the coefficient M_{max}/M increases. The relative difference between the K-L divergence of the approximate and the random distributions suggests that for values of M_{max}/M above 0.5 the approximate distribution provides a good fit to the exact solution.

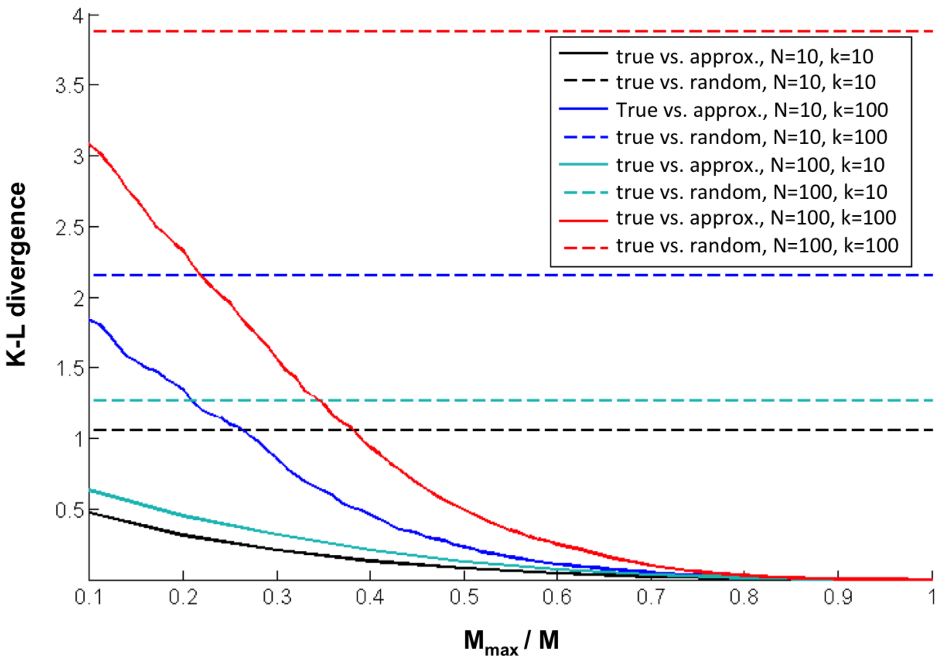

Figure 1. Kullback-Leibler divergence between the true and the approximate likelihood function, $\lambda(X)$. This scenario simulates a set of generic child node sending λ messages to its parent node in order to measure how well our proposed method approximates the true result. Results are shown for different values of the number of incoming messages used (M_{max}), the total number of incoming messages (M), and the number of states (k). Results are averaged over 500 trials. The Kullback-Leibler (K-L) divergence, on the y-axis, measures the cross-correlation (similarity) between an approximate distribution and the true distribution. The dotted lines show the K-L divergence between the true and a random distribution, which serves as a baseline to compare the goodness of fit of the approximate distribution.

To check how well the sampling procedure (Equations (7) and (8)) managed to approximate the exact belief functions (Equations (3) and (4)), the method was also tested statistically by simulating a generic child node, X, receiving messages from its parent nodes, $U_1, .., U_N$. Using randomly generated CPTs $P(X|U_1, \cdots, U_N)$ and likelihood functions $\lambda(x)$, the difference between the exact beliefs and the approximated beliefs obtained after sampling was measured using the Kullback-Leibler (K-L) divergence.

Figure 2 shows the K-L divergence between the real and approximate beliefs, averaged over 100 trials, as a function of N_{max} and k_{umax}. For comparison, the K-L divergence between the exact belief and a randomly generated belief distribution is also plotted. The range over which these parameters are tested is limited by the computational cost associated with calculating the exact beliefs using CPTs of size exponential to the number of parents. Thus the chosen parameters are $k_X = 4, k_U = 10, N = 6, K_{umax} = \{1 \cdots 10\}$ and $N_{max} = \{1 \cdots 6\}$.

The results show that as N_{max} and k_{umax} increase, the goodness of fit between the approximation and the exact belief increases. Furthermore, the relative difference between the K-L divergence of the approximate and the random belief distributions suggests that even for relatively small values of N_{max} and k_{umax} the approximate belief provides a good fit to the exact belief.

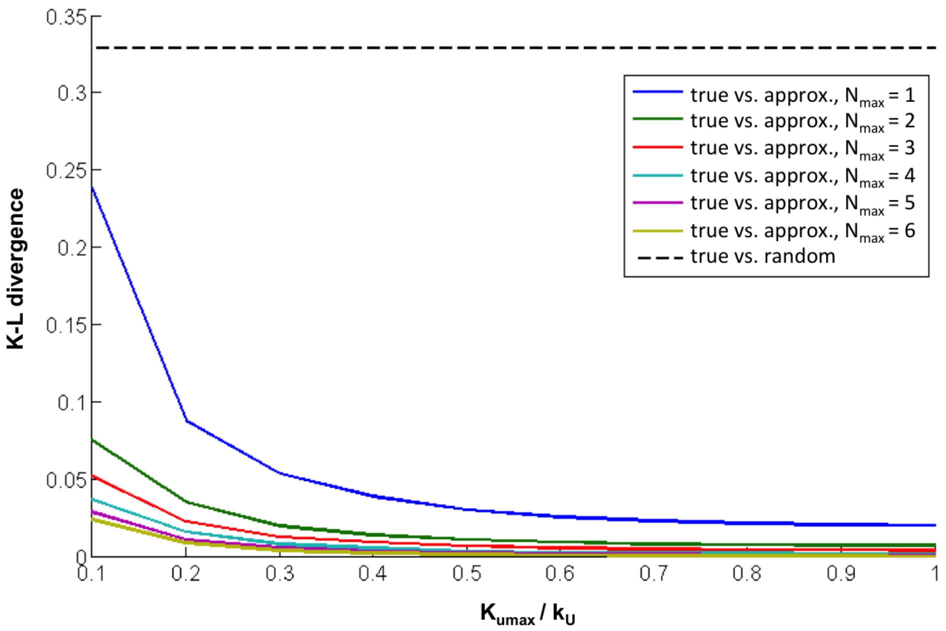

Figure 2. Kullback-Leibler divergence between the true and the approximate prior function, $\pi(X)$. This scenario simulates a generic child node receiving π messages from its parent nodes in order to measure how well our proposed method approximates the true result. Results are shown for different values of the number of samples taken from each π messages (k_{umax}) and the number of parents used (N_{max}), averaged over 100 trials. Results are plotted for values of N_{max}, ranging from 1 to 6 as indicated in the colour legend. The Kullback-Leibler (K-L) divergence, on the y-axis, measures the cross-correlation (similarity) between an approximate distribution and the true distribution. The dotted horizontal lines shows the K-L divergence between the true and a random distribution, which serves as a baseline to compare the goodness of fit of the approximate distributions.
